# Single-cell capture of on-ART SIV transcription reveals TGF-**β**–mediated metabolic control of viral latency

**DOI:** 10.1172/jci.insight.198810

**Published:** 2026-02-23

**Authors:** Romaila Abd-El-Raouf, Jakob Harrison-Gleason, Jinhee Kim, Ching Man Wai, Kayla L. Yerlioglu, Catarina Ananias-Saez, Alec Ksiazek, Jeffrey T. Poomkudy, Mariluz Araínga, Deepanwita Bose, Claudia Cicala, James Arthos, Francois J. Villinger, Ramon Lorenzo-Redondo, Elena Martinelli

**Affiliations:** 1 Department of Medicine, Division of Infectious Diseases, and; 2 Department of Biochemistry and Molecular Genetics, Feinberg School of Medicine, Northwestern University, Chicago, Illinois, USA.; 3 New Iberia Research Center, University of Louisiana at Lafayette, New Iberia, Louisiana, USA.; 4 Laboratory of Immunoregulation, National Institute of Allergy and Infectious Diseases, National Institutes of Health, Bethesda, Maryland, USA.; 5 Center for Pathogen Genomics and Microbial Evolution, Northwestern University Havey Institute for Global Health, Chicago, Illinois, USA.; 6 Department of Microbiology and Immunology, Feinberg School of Medicine, Northwestern University, Chicago, Illinois, USA.

**Keywords:** AIDS/HIV, Immunology, Metabolism, Cellular immune response, T cells

## Abstract

We previously demonstrated that blocking TGF-β with galunisertib, a safe, orally available small drug, reactivated latent SIV in vivo by shifting T cells toward a transitional effector phenotype. Here, we investigated the mechanisms underlying this effect using single-cell RNA sequencing, metabolic profiling, and high-dimensional spectral flow cytometry of samples from SIV-infected, antiretroviral therapy–treated (ART-treated) macaques before and after galunisertib. To characterize virus-transcribing, infected cells during ART, we developed a novel, sensitive SIV Transcripts Capture Assay (SCAP) that detected 127 SIV-expressing cells within lymph node single-cell transcriptome libraries. Galunisertib drove broad metabolic reprogramming in CD4^+^ T cells, with transcriptional upregulation of inflammatory and mitochondrial biosynthesis pathways, confirmed by Seahorse profiling. Metabolomics revealed increased energy metabolites and amino acids and enhanced metabolic flux without proliferation. SIV transcript–positive cells before galunisertib were metabolically quiescent compared with cells without detectable viral transcripts. After galunisertib, virus-expressing cells showed a dramatic metabolic activation, with upregulation of glycolysis, fatty acid metabolism, and TNF-α signaling. High-dimensional flow cytometry demonstrated effects beyond CD4^+^ T cells, including fewer tissue-resident memory T cells, but more inflammatory macrophages. In conclusion, SCAP represents a specific tool for characterizing rare SIV-infected cells transcribing virus during ART, and it reveals TGF-β as a key mediator of viral latency in vivo through metabolic suppression.

## Abstract 2

Characterization of cells producing virus in tissues in macaques with SIV on therapy points to metabolic reprogramming as a way to reverses SIV latency.

## Introduction

HIV and SIV establish persistent viral reservoirs that represent major obstacles to cure strategies (1, 2). These reservoirs consist of infected cells harboring proviruses that persist despite antiretroviral therapy (ART) (3). Viral transcription continues from both defective and intact proviruses, with the immune system playing a crucial role in containing these on-ART reactivation events and shaping reservoir composition (4). This dynamic interplay, alongside heterogeneous latency mechanisms, complicates reservoir elimination efforts.

Elevated TGF-β levels have been documented in people living with HIV (PLWH) since the early epidemic (5), and remain high despite prolonged ART, correlating with immune dysfunction and disease progression (6–8). While TGF-β’s immunosuppressive and fibrogenic properties have been well characterized in the context of HIV (9, 10), TGF-β’s direct impact on HIV infection and transcription remains under-characterized. This knowledge gap is significant considering TGF-β’s central role in primary CD4^+^ T cell latency models (11–13) and its regulation of immune processes influencing viral persistence (14, 15).

Our previous work demonstrated that TGF-β signaling contributes to the maintenance of viral latency and that inhibition of this pathway using galunisertib (LY2157299), a small, safe, orally available drug that reached phase II clinical development with Eli Lilly (16, 17), can effectively reverse latency in SIV-infected rhesus macaques on ART (18, 19). Specifically, we showed that TGF-β blockade induced latency reversal ex vivo in cells from PLWH and in vivo in SIV-infected, ART-treated rhesus macaques (18, 19). Moreover, we connected the in vivo latency reversal with the induction of a transitional effector phenotype in CD4^+^ T cells characterized by an increase in some, but not all, canonical activation markers at both the transcriptional and the protein level (19). Finally, in vivo galunisertib treatment also led to enhanced SIV-specific responses and attrition of the viral reservoir (19).

Notably, in previous studies, we consistently observed a substantial enrichment of metabolic pathways in T cells from blood and lymph nodes. Pathways linked to mitochondrial biosynthesis and respiration were significantly upregulated following galunisertib treatment in vivo in both infected and uninfected macaques (18, 19).

The relationship between HIV latency and cellular metabolism has emerged as a critical determinant of viral persistence (20–22). HIV preferentially infects metabolically active CD4^+^ T cells (22, 23), and upon ART initiation, infected cells that survive likely transition to a quiescent metabolic state, contributing to the establishment of the long-lived reservoir (22, 24). This metabolic quiescence is characterized by reduced glycolysis, oxidative phosphorylation, and overall decreased energy production — an environment unfavorable for viral transcription and production (25).

Recent studies have demonstrated that manipulating specific metabolic pathways can effectively reverse HIV latency (26). For instance, activators of mTOR signaling stimulate glycolysis and anabolic metabolism, leading to increased viral transcription (21, 27). Similarly, compounds that enhance mitochondrial function have shown promise in reversing latency in various models (28, 29). TGF-β signaling itself is intimately connected to cellular metabolism, as TGF-β promotes metabolic quiescence in various immune cells, particularly T cells, by suppressing glycolysis and mitochondrial activity (30–32).

Here, we hypothesized that the main mechanism underlying the latency reversal effect seen in vivo by TGF-β blockade would be connected to its impact on cellular metabolism. To test this hypothesis, we developed a highly sensitive way to characterize SIV-infected cells producing viral transcripts on ART before and after treatment.

Conventional approaches to detect viral RNA–positive cells within single-cell transcriptome studies have typically been based on the identification of cells expressing viral transcripts bioinformatically, leading to potential biases toward high-expressing cells and dependence on sequencing depth (33, 34). We developed a sensitive SIV transcripts capture assay (SIV Transcripts Capture Assay with Parse Biosciences [SCAP]) that enabled a less biased identification and characterization of SIV RNA–positive cells within single-cell RNA sequencing (scRNA-seq) data from lymph node cells before and after galunisertib treatment. Using SCAP, accompanied by metabolomics and Seahorse studies on bulk CD4^+^ T cells, we demonstrated that galunisertib-mediated latency reversal is predominantly driven by enhanced cellular metabolism. Moreover, high-dimensional flow cytometry analyses revealed that this metabolic reprogramming extended beyond CD4^+^ T cells to include other immune cell populations in tissues, such as macrophages and NK cells.

## Results

### In vivo TGF-β blockade increases lymph node CD4^+^ T cell metabolism.

**Figure 1:**
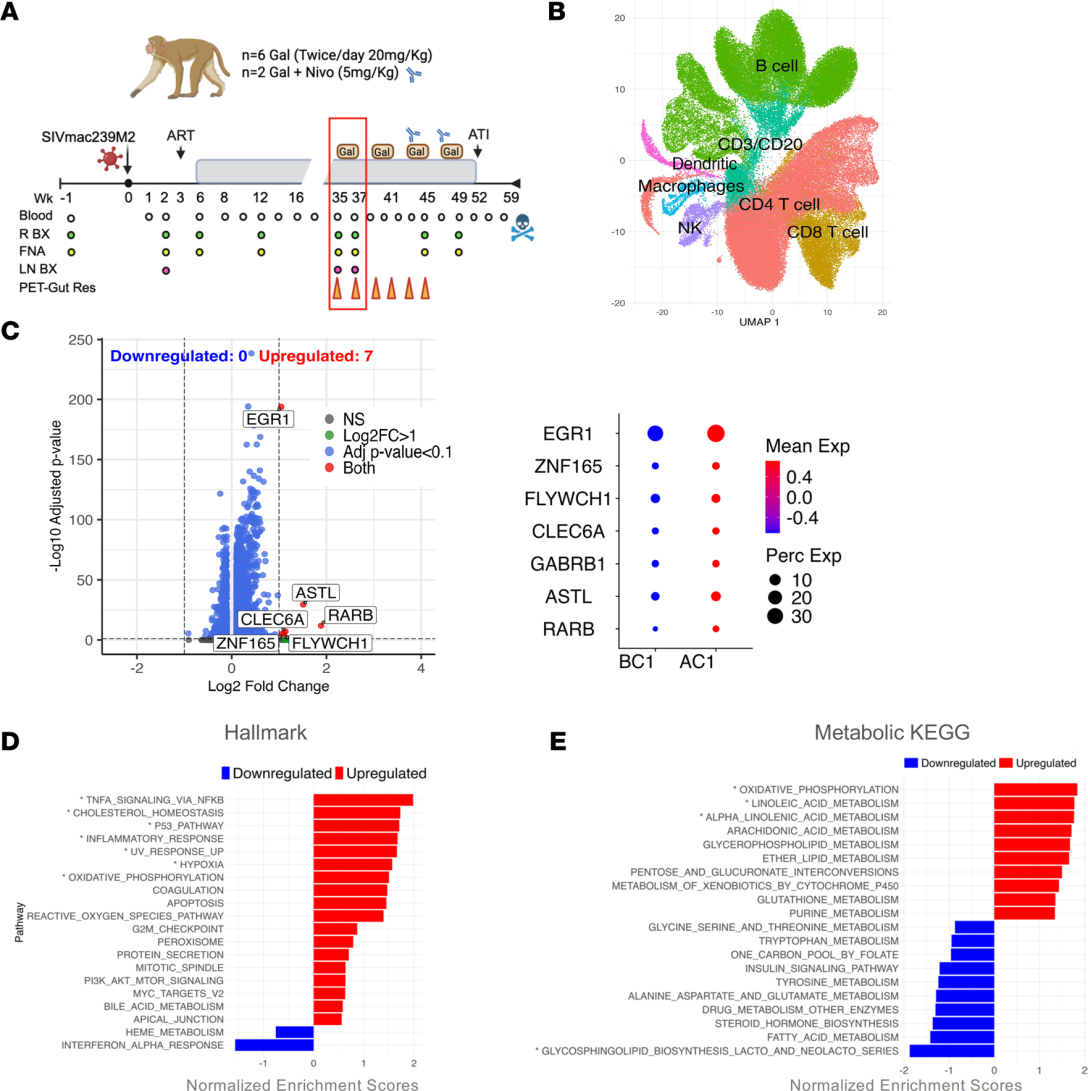
Galunisertib upregulates metabolic pathways in lymph node CD4^+^ T cells. (**A**) Schematic of the study: Eight macaques were infected with SIVmac239M2 intravenously, and ART was started at week 6 post-infection. The red rectangle indicates the time points analyzed by scRNA-seq: before (BC1; week 35 post-infection) and after the first galunisertib (Gal) cycle (AC1; week 37 post-infection) when the lymph nodes were collected. LN BX, lymph node biopsy; R BX, rectal biopsy; ATI, Antiretroviral therapy interruption; FNA, fine-needle aspiration; Nivo, nivolumab. (**B**) UMAP projection of scRNA-seq data from lymph node cells, showing annotation of distinct cell subsets. (**C**) Volcano plot showing differentially expressed genes (DEGs) resulting from the comparison of CD4^+^ T cells by MAST hurdle model at BC1 versus AC1. Labeled genes that reached significance (BH FDR–adjusted *q* ≤ 0.1) and log_2_FC ≥ 1 are shown in the bubble plot. (**D** and **E**) Enriched hallmark (**D**) and KEGG metabolic (**E**) pathways in total CD4^+^ T cells based on GSEA (BC1 vs. AC1). Upregulated pathways (red) and downregulated pathways (blue) are shown with their respective normalized enrichment scores (BH FDR *q ≤ 0.1).

This study builds on previously published in vivo research (Figure 1A) wherein eight SIVmac239M2-infected, ART-treated rhesus macaques received four 2-week cycles of galunisertib (20 mg/kg, orally, twice daily). The original investigation demonstrated that galunisertib induced a transitional effector phenotype in CD4^+^ T cells, leading to on-ART latency reversal, enhanced immune responses, and reduced SIV reservoirs. Latency reversal was evidenced by increased plasma and cell-associated viral RNA, as well as by immunoPET/CT, which revealed substantial viral reactivation in deep tissue compartments (19).

To elucidate the mechanistic impact of TGF-β signaling blockade in vivo with galunisertib, we conducted a targeted reanalysis of scRNA-seq data generated from lymph node cells of the 8 macaques collected before (BC1) and after (AC1) the first 2-week galunisertib cycle (Figure 1A). The scRNA-seq data were clustered using the Louvain algorithm and visualized with uniform manifold approximation and projection (UMAP) (35) to reduce dimensionality and display transcriptional heterogeneity. Clusters were annotated with identified cell types (Figure 1B) based on canonical marker gene expression (Supplemental Figure 1A; supplemental material available online with this article; https://doi.org/10.1172/jci.insight.198810DS1).

Differential expression analysis of the CD4^+^ T cell cluster before (BC1) and after (AC1) galunisertib revealed 7 significantly upregulated genes with no downregulated genes (Benjamini-Hochberg [BH] FDR–adjusted *q* < 0.1; Figure 1C). These differentially expressed genes (DEGs) are implicated in cellular activation (*EGR1*, *ZNF165*, *FLYWCH1*) (36, 37), differentiation, migration, and immune recognition (*RARB*, *CLEC6A*, *ASTL*) (38, 39), confirming the ability of TGF-β blockade to drive immune activation and T cell differentiation. Gene set enrichment analysis (GSEA) further confirmed robust upregulation of hallmark activation and inflammatory pathways, lipid homeostasis, and stress responses (Figure 1D).

Notably, consistent with previous studies in which oxidative phosphorylation (OXPHOS) was among the most enriched pathways after galunisertib (18, 19), we observed significant upregulation of genes involved in OXPHOS and mitochondrial respiration, along with overall enrichment in metabolic pathways (Figure 1D and Supplemental Figure 1B). To characterize the specific impact of TGF-β blockade on CD4^+^ T cell metabolism, we performed GSEA on a curated set of metabolic pathways within the Kyoto Encyclopedia of Genes and Genomes (KEGG) database (Supplemental Table 1). Corroborating the hallmark results, this analysis revealed strong upregulation of OXPHOS following galunisertib, reinforcing the observation of enhanced mitochondrial activity. Additionally, proinflammatory lipid metabolism pathways were upregulated, including linoleic, arachidonic, and α-linolenic acid pathways (Figure 1E and Supplemental Figure 1C).

Conversely, several amino acid metabolic pathways — glycine, serine, threonine, tryptophan, tyrosine, and glutamate — trended toward downregulation, indicating a shift away from biosynthetic maintenance typically associated with quiescent T cells. This metabolic profile suggests a transition toward a transcriptionally engaged, metabolically active state rather than one primed for proliferation. Supporting this increased energetic demand in the absence of enhanced proliferation, we observed downregulation of one-carbon pool by folate and reduced glycosphingolipid biosynthesis, processes typically upregulated during proliferation to support cell division and expansion (40, 41). Collectively, these findings demonstrate comprehensive metabolic reprogramming favoring effector function, stress adaptation, and transcriptional readiness in CD4^+^ T cells following galunisertib treatment.

**Figure 2:**
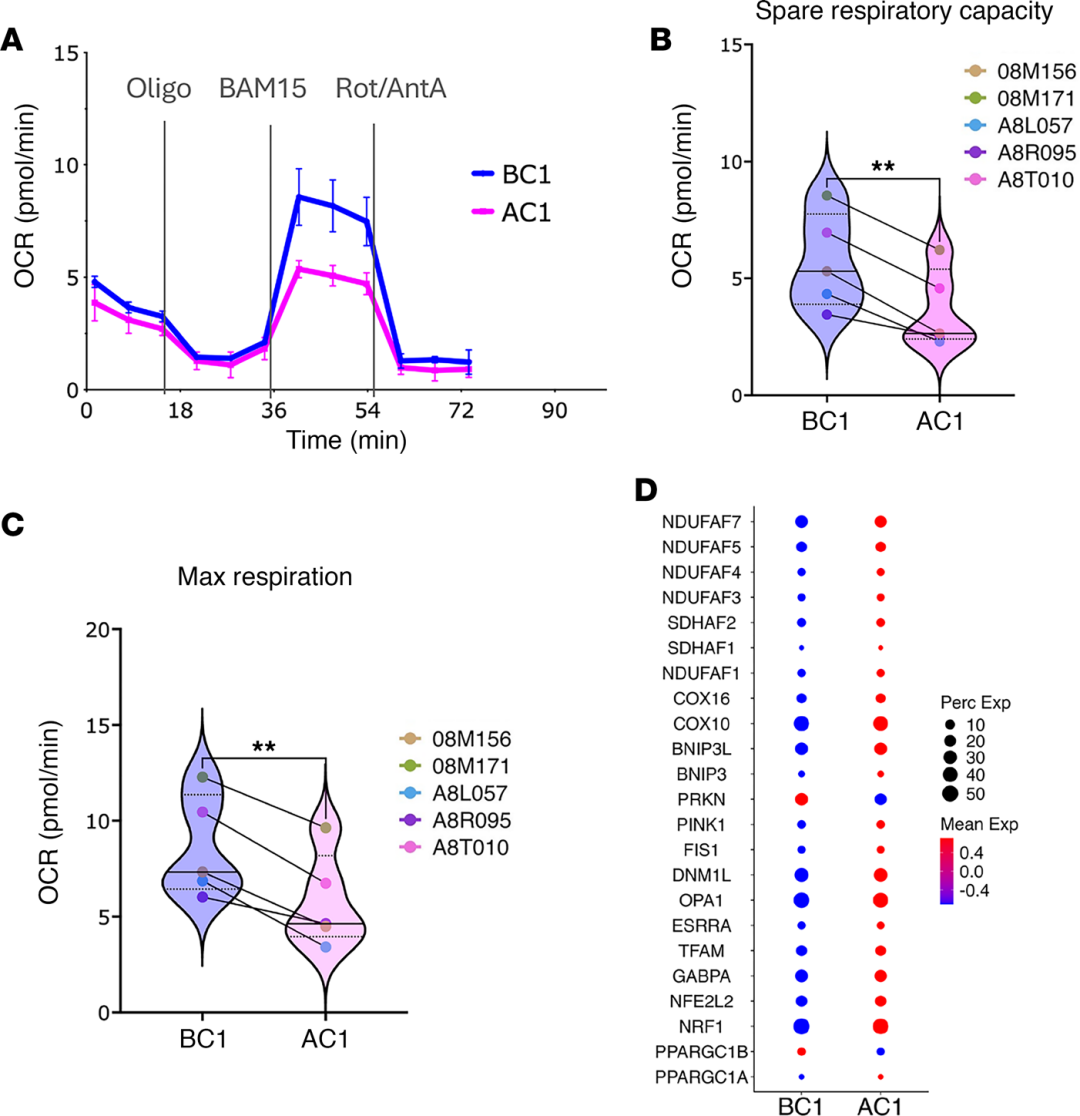
Galunisertib impacts lymph node CD4^+^ T cell metabolism. (**A**) Representative Seahorse T cell metabolic profiling of CD4^+^ T cells isolated from the lymph nodes of a monkey (08M156) before (BC1; week 35 post-infection) and after galunisertib (AC1; week 37 post-infection), showing basal oxygen consumption rate (OCR) and changes in OCR due to response to oligomycin (complex V inhibitor), BAM15 (mitochondrial uncoupler), and rotenone and antimycin A (inhibitors of mitochondrial complex I/III). (**B** and **C**) Spare and maximal respiratory capacity calculated from Seahorse T cell metabolic profiling of CD4^+^ T cells isolated from the lymph nodes of 5 macaques before (BC1) and after (AC1) galunisertib. Data were normally distributed (Shapiro-Wilk test) and compared by paired 2-tailed *t* test (**P ≤ 0.01). (**D**) The relative expression before and after galunisertib of selected genes associated with mitochondrial biogenesis and function (Supplemental Table 2).

To better understand the metabolic reprogramming driven by TGF-β blockade, we sorted CD4^+^ T cells from the remaining lymph node cells of 5 of the 8 macaques before and after the first galunisertib cycle and tested their metabolic output with a Seahorse T cell metabolic profiling assay. As suggested by the transcriptomics data, CD4^+^ T cells after galunisertib were more metabolically active than those from before treatment. Specifically, following galunisertib, we observed a significant reduction in the cells’ spare and maximal respiratory capacity (RC), confirming a reduced ability to respond to increased energy demands by increasing their mitochondrial RC (Figure 2, A–C). Reduced spare and maximal RC accompanied by a tendency toward reduced basal oxygen consumption rate (Supplemental Figure 1D) is typical of effector cell subsets, confirming our previous description of these cells as being in a transitional effector state. However, to reconcile the transcriptional increase in OXPHOS genes, typically associated with quiescence in T cells (34), with the increased energy metabolism and effector phenotype of these cells, we focused our transcriptional analysis on key genes involved in mitochondrial biosynthesis included in the OXPHOS hallmark and KEGG pathways (Supplemental Table 2). We observed increased expression of all genes specifically involved in mitochondrial biosynthesis and assembly, including *PPARGC1A*, *NRF1*, *NFE2L2*, *TFAM*, and *GABPA* (42–44), and several associated with mitochondrial fission and fusion, including *OPA1*, *FIS1*, *DNM1L* (DRP1) (45) and *ESRRA* (46) (Figure 2D). This analysis suggests that the observed increase in the OXPHOS pathway reflects cellular adaptation to increased energetic demand rather than a direct increase in OXPHOS, which is usually associated with T cell memory maintenance and stemness (47, 48).

Collectively, these findings suggest that in CD4^+^ T cells, the primary effect of TGF-β blockade was metabolic reprogramming, with pronounced upregulation of metabolic pathways associated with cellular activation and effector function. By contrast, while CD8^+^ T cells also exhibited enhanced energetic metabolism following galunisertib, their transcriptional response was more robust (higher number of DEGs at *q* < 0.1) and predominantly driven by upregulation of inflammatory and effector function genes rather than metabolic pathway genes (Supplemental Figure 2).

### Higher metabolic signature explains post–TGF-β blockade viral transcription.

To determine how galunisertib’s transcriptional and metabolic reprogramming in CD4^+^ T cells connected with SIV latency reversal, we focused our transcriptomic analysis on cells expressing viral transcripts. To identify cells expressing SIV transcripts in our scRNA-seq dataset in a specific and sensitive manner, we developed a novel technique termed SIV Transcripts Capture Assay with Parse Biosciences (SCAP; Supplemental Figure 3A).

We designed a panel of 314 probes targeting the SIV coding region, covering each coding DNA sequence (CDS) with 4 different, overlapping probes. Each probe was designed to avoid exon boundaries to prevent issues with differential splicing. For shorter CDS regions, like *tat* and *rev*, we designed 2 full-length probes bookended from the start or stop and extended up- and downstream. After probe hybridization, SIV transcripts were amplified and sequenced, generating an SIV transcript–enriched library that retained cell- and sample-specific barcodes and could be integrated with the whole-transcriptome library. This technique successfully captured viral transcripts with excellent coverage, particularly within the *gag* region (Supplemental Figure 3B). In contrast, a negligible number of SIV reads were identified in the whole-transcriptome library before SCAP (Supplemental Figure 3B).

**Figure 3:**
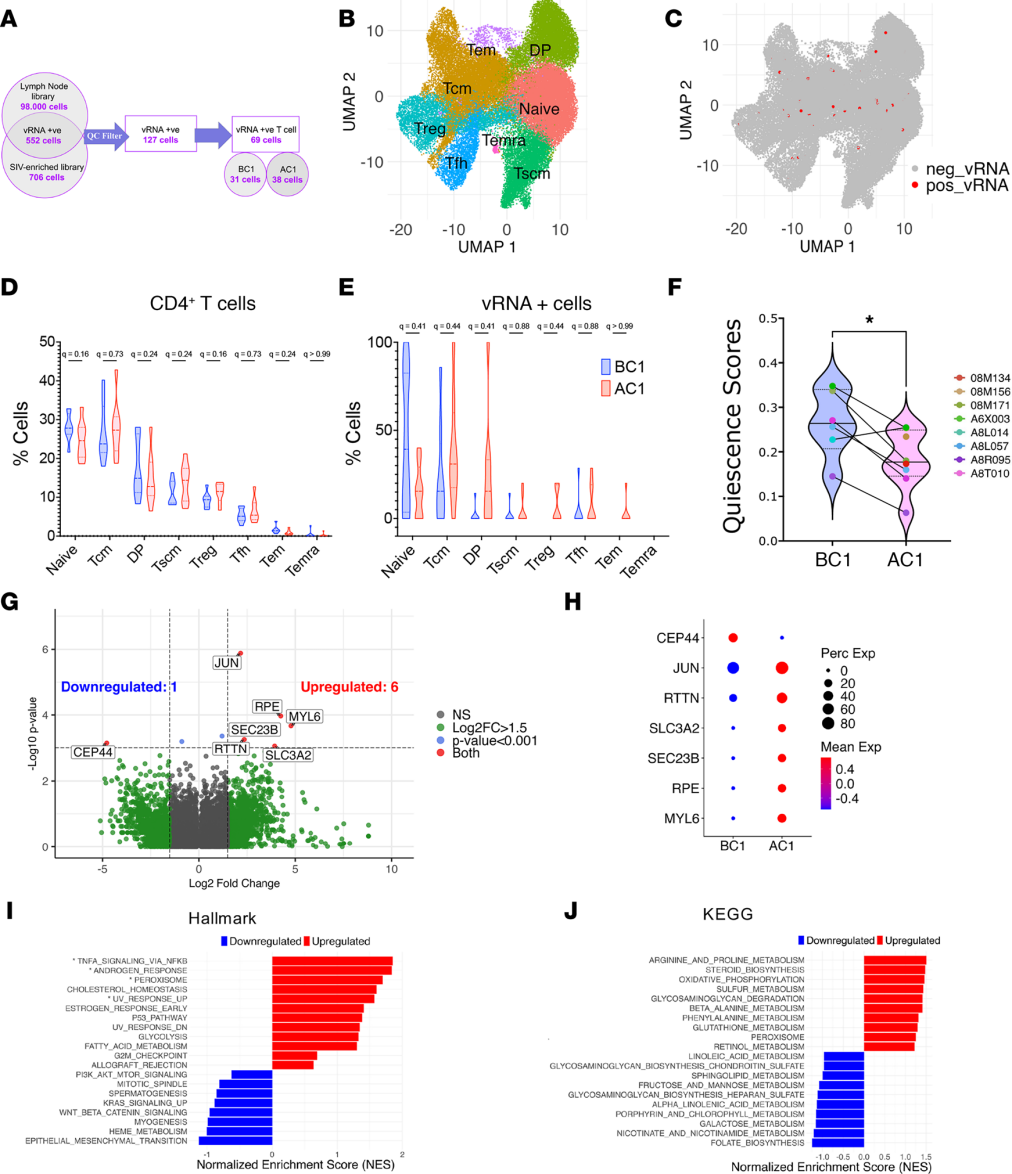
Lymph node cells transcribing SIV on ART display a higher metabolic signature after galunisertib. (**A**) Schematic process for the identification and filtering of vRNA-positive cells from merged lymph node whole-transcriptome library (98,000 cells) and the SCAP analysis (which yielded 706 cells). Five hundred fifty-two cells had a corresponding barcode in the whole-transcriptome library analyzed with standard Parse pipeline. Only 127 cells passed QC (features between 50 and 8,000 and total UMI between 450 and 200,000). (**B** and **C**) UMAP projection of the CD4^+^ T cell cluster from Figure 1 showing manual annotation of distinct cell subsets (**B**) and highlighted SIV transcript–positive cells (vRNA^+^) (**C**). (**D** and **E**) The frequencies of cells, CD4^+^ T cells (**D**) and vRNA^+^ (**E**), in each CD4^+^ T cell subset before (BC1) and after (AC1) galunisertib are shown compared by negative binomial mixed-effects models and BH FDR correction. (**F**) Comparison of quiescence module scores calculated on genes included in Supplemental Table 3 in SIV^+^ cells before (BC1) and after (AC1) galunisertib by Wilcoxon signed-rank test (*P ≤ 0.05). (**G**) Volcano plot of DEGs in vRNA^+^ T cells (AC1 vs. BC1) using MAST hurdle model (*P* ≤ 0.001; abs log_2_FC ≥ 1.5). Labeled genes have a *P* less than or equal to 0.001. (**H**) Bubble plot showing DEGs with a differential expression of abs log_2_FC ≥ 1.5 and *P* ≤ 0.001. (**I** and **J**) Enriched hallmark (**I**) and KEGG metabolic (**J**) pathways in vRNA^+^ CD4^+^ T cells based on GSEA (BC1 vs. AC1). (**I**) Asterisks indicate pathways reaching FDR-adjusted significance (q < 0.1).

Integration of whole-transcriptome and SCAP data revealed 552 candidate SIV RNA–positive (vRNA^+^) cells. In contrast, no vRNA^+^ cells were detected in our negative control sample (uninfected PBMCs) subjected to the same SCAP pipeline. Hence, all 552 SIV-RNA^+^ cells from SCAP of SIV-infected samples were considered. After quality control (QC) filtering to remove low-quality cells, 127 high-confidence SIV-RNA^+^ cells were retained (Figure 3A).

Notably, before QC, the number of SIV-RNA^+^ cells was significantly higher after galunisertib than before (345 SIV-RNA^+^ cells from 1,015,879 SIV-RNA^–^ total barcodes compared with 207 SIV-RNA^+^ cells from 1,206,103 SIV-RNA^–^ initial total barcodes; χ^2^ with Yates’s correction *P* < 0.001). However, the difference did not reach significance after QC (68 SIV-RNA^+^ cells and 45,947 SIV-RNA^–^ cells compared with 59 SIV-RNA^+^ cells and 51,844 SIV-RNA^–^ cells; Fisher’s exact test *P* = 0.154).

Among the 127 high-confidence SIV-RNA^+^ cells, 69 were identified as T cells, including 31 from before and 38 from after galunisertib treatment (Figure 3A). To investigate the phenotype of vRNA^+^ T cells, we reclustered the CD4^+^ T cell population into distinct subsets using canonical marker gene expression (Figure 3B and Supplemental Figure 3). As shown in Figure 3C, most vRNA^+^ T cells belonged to memory subsets, with a substantial number in the naive cluster as well. Few vRNA^+^ cells belonged to the T stem cell memory (TSCMs) or Treg clusters. When comparing subset distribution before (BC1) and after (AC1) galunisertib at the global level, we observed a small reduction in naive cells and an increase in Tregs and Tscm cells, which was not significant after multiple comparisons (negative binomial mixed-effects models; BH FDR *q* > 0.1; Figure 3D). Conversely, among vRNA^+^ cells, we observed fewer cells in the naive cluster and higher frequencies in memory clusters after galunisertib (Figure 3E). However, the changes did not reach significance (BH FDR *q* > 0.1), likely because of the complete absence of viral transcript–positive cells in macaques 08M134 and 08M156 before galunisertib (BC1). Moreover, all other macaques, except A6X003, had vRNA^+^ cells only within naive or central memory T cell clusters before galunisertib. In contrast, after galunisertib (AC1), macaques 08M134 and 08M156 had vRNA^+^ cells in all clusters, and all other macaques had a decrease in vRNA^+^ cells within naive, while all memory clusters became populated without a specific enrichment in a single cluster.

To further assess the activation state of vRNA^+^ cells, we calculated a quiescence score based on a curated set of genes associated with T cell quiescence (Supplemental Table 3). The quiescence scores — reflecting transcriptional programs associated with a quiescent-like state — in vRNA^+^ cells significantly decreased (*P* ≤ 0.05) after galunisertib treatment, supporting the increased metabolic activity of vRNA^+^ cells after treatment (Figure 3F).

Upon comparing gene expression profiles of vRNA^+^ cells before (BC1) and after (AC1) galunisertib, we identified 6 putative upregulated genes and 1 putative downregulated gene (uncorrected *P* < 0.001; absolute log_2_ fold change [log_2_FC] > 1.5; Figure 3G). Among them, upregulation of *RPE* (pentose phosphate pathway), *SEC23B* (protein transport), *SLC3A2* (amino acid transport), *MYL6* (myosin light chain), and *JUN* (transcription factor) points to a shift toward increased energy metabolism and a transcriptionally permissive state (Figure 3H). In contrast, downregulation of *CEP44* (centrosomal protein) suggests reduced proliferation, in line with the ability of TGF-β blockade to uncouple cellular activation from cellular proliferation noted above. Notably, only *JUN* reached significance after multiple-testing correction (BH FDR *q* ≤ 0.1) (Figure 3H).

GSEA confirmed a coordinated shift of vRNA^+^ cells toward increased metabolism, activation, and stress adaptation following galunisertib (Figure 3I). Key transcriptional programs upregulated after treatment included TNF-α signaling via NF-κB (highest normalized enrichment score), androgen response, peroxisome function, cholesterol homeostasis, UV response, and estrogen response pathways. Conversely, pathways associated with epithelial-mesenchymal transition, heme metabolism, myogenesis, and WNT/β-catenin signaling trended toward downregulation. This enrichment pattern suggests a fundamental shift toward an activated, metabolically energized cellular state.

Enrichment analysis within the hallmark (Figure 3I and Supplemental Figure 4A) and KEGG (Figure 3J and Supplemental Figure 4B) sets provided complementary evidence of metabolic reprogramming. Hallmark analysis showed enrichment of glycolysis and fatty acid metabolism, while the KEGG metabolic-focused analysis revealed a significant upregulation of OXPHOS and a trend toward enrichment of multiple other metabolic pathways, including arginine and proline metabolism, steroid biosynthesis, and peroxisome function. Together, these enrichment patterns point to increased and diversified metabolic output as the main differentiating factor in cells producing viral transcripts after galunisertib.

### TGF-β blockade reverses metabolic quiescence in infected cells to reinitiate viral transcription.

**Figure 4:**
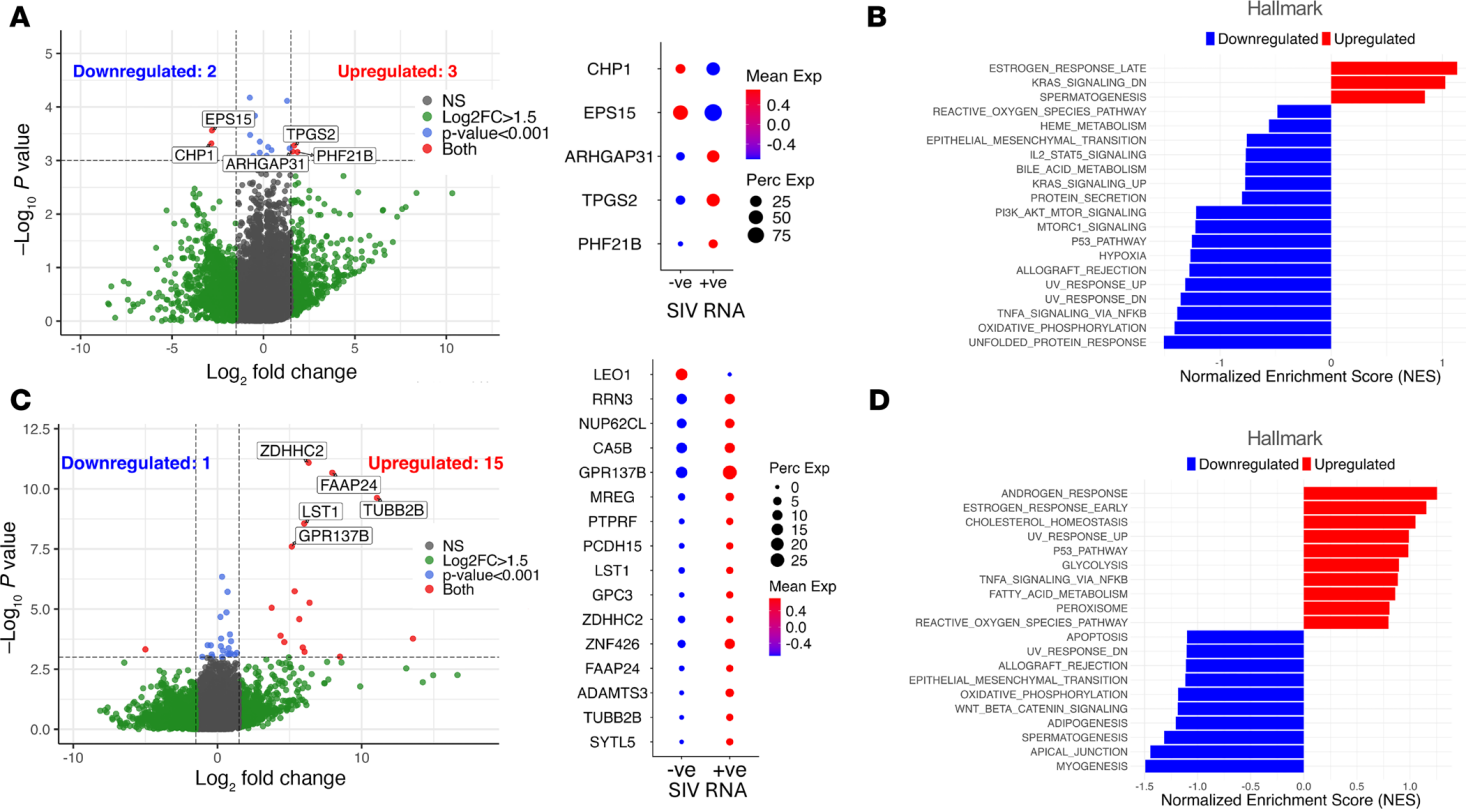
TGF-β blockade reverses metabolic quiescence in infected cells producing viral transcripts on ART. (**A**) Volcano plot of DEGs resulting from the comparison of vRNA^+^ versus vRNA^-^ T cells before galunisertib (BC1) treatment (BC1; *P* ≤ 0.001; abs log_2_FC ≥ 1.5) and bubble plot of DEGs with *P* ≤ 0.001 and abs log_2_FC ≥ 1.5. (**B**) Enriched hallmark pathways in vRNA^+^ compared with vRNA^–^ T cells before galunisertib (BC1). (**C**) Volcano plot of DEGs resulting from comparison of vRNA^+^ versus vRNA^–^ CD4^+^ T cells by MAST hurdle model after the first cycle of galunisertib treatment (AC1, *P* ≤ 0.001; abs log_2_FC ≥ 1.5) and bubble plot of DEGs (*P* ≤ 0.001 and abs log_2_FC ≥ 1.5). (**D**) Enriched hallmark pathways in vRNA^+^ compared with vRNA^–^ CD4^+^ T cells after galunisertib (AC1). (BH FDR *q* ≤ 0.1.)

To understand whether viral transcription was associated with enhanced cellular metabolism independent of galunisertib, we compared the transcriptomes of vRNA^+^ T cells versus vRNA^–^ T cells at baseline, before treatment. Differential expression analysis identified 3 putatively upregulated and 2 downregulated genes in SIV-RNA^+^ cells (*P* ≤ 0.001 and log_2_FC > 1.5; BH FDR > 0.1; Figure 4A). Among them, the upregulation of *PHF21B*, a transcriptional and epigenetic repressor (49), together with the downregulation of *CHP1* and *EPS15*, which control, respectively, calcium signaling through NFAT (50) and T cell receptor endocytosis, strongly suggests a quiescence/exhausted phenotype. Notably, none of the putative DEGs reached significance at BH FDR < 0.1, suggesting that we did not have enough power to detect significant differences with the very small number of vRNA^+^ cells.

GSEA confirmed a quiescent phenotype in vRNA^+^ cells before galunisertib by revealing a tendency toward OXPHOS, PI3K/AKT/mTOR signaling, and mTORC1 downregulation in the virus-expressing cells compared with uninfected cells or infected cells not transcribing the virus (Figure 4B and Supplemental Figure 4C). Inflammatory pathways, including TNF-α signaling via NF-κB, and cellular stress response pathways such as unfolded protein response also trended toward downregulated in vRNA^+^ compared with vRNA^–^ cells before galunisertib. This pattern suggests a reduced activation status in cells transcribing the virus during ART compared with uninfected cells in the absence of any intervention.

In contrast, when we compared vRNA^+^ T cells with vRNA^–^ cells after galunisertib, we found 15 putatively upregulated genes and 1 putatively downregulated gene (*P* ≤ 0.001; BH FDR > 0.1; log_2_FC > 1.5; Figure 4C). Six of the upregulated DEGs (*TUBB2B*, *FAAP24*, *ZDHHC2*, *LST1*, *MREG*, *GPR137B*) reached significance after multiple-comparison correction (BH FDR–adjusted *q* ≤ 0.1), demonstrating a larger overall difference between infected cells transcribing virus after galunisertib and cells without detectable viral transcripts. Some of these upregulated DEGs, including *FAAP24*, *ZDHHC2*, and *LST1*, point to enhanced stress responses and DNA repair (51, 52). Other DEGs with high differential expression point to altered mitochondrial metabolism (*CA5B*) (53), modified endosomal dynamics (*GPR137B*) (54), and increased ribosomal RNA synthesis (*RRN3*) (55), suggesting increased protein synthesis and processing. Altogether, these gene expression changes suggest that viral transcription after galunisertib, but not before, is associated with a higher metabolic state and increased transcription, translation, and stress adaptation.

Pathway enrichment analysis (Figure 4D and Supplemental Figure 4D) displayed a shift toward metabolic and inflammatory programs in post-treatment vRNA^+^ cells. Among the most highly enriched pathways that trended toward upregulation were androgen response, early estrogen response, cholesterol homeostasis, and UV response. Notably, glycolysis, TNF-α signaling via NF-κB, and fatty acid metabolism pathways also trended toward upregulation, pointing to enhanced energy metabolism and lipid processing. Conversely, multiple quiescence-associated pathways, including apoptosis, UV response, epithelial-mesenchymal transition, and OXPHOS, trended toward downregulation.

Altogether, these gene expression changes suggest that while viral transcription in ART-treated macaques is associated with a quiescent metabolic state, TGF-β blockade reverses this pattern, promoting higher metabolic activity and increased transcription/translation machinery that contributes to increase viral transcription.

### TGF-β blockade increases metabolism of blood immune cells.

**Figure 5:**
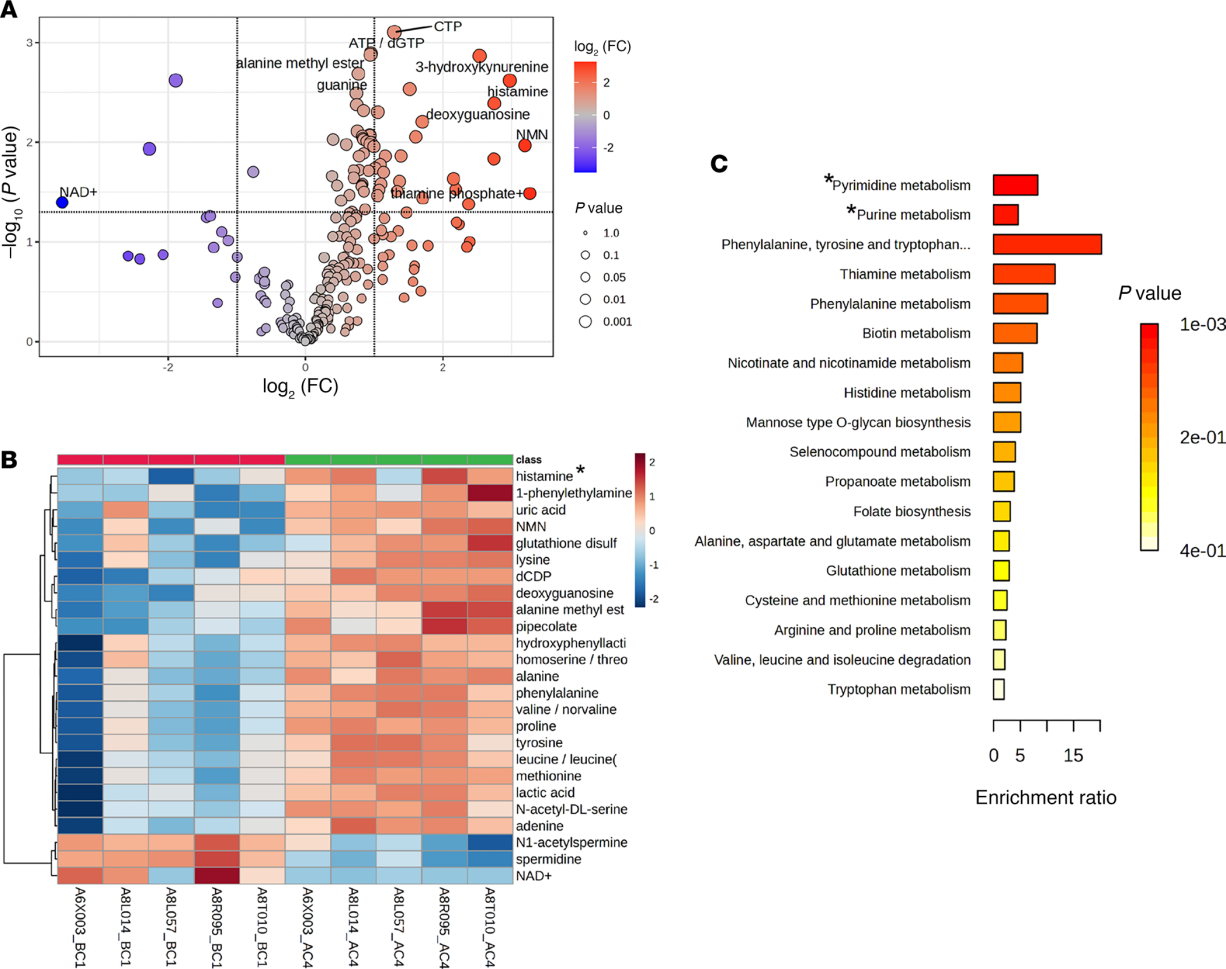
TGF-β blockade increases metabolism of blood immune cells. Metabolites were extracted from the pellets of PBMCs collected right before (BC1, before cycle 1; week 35 post-infection) and after (AC4, after cycle 4; week 49 post infection) all four 2-week cycles with galunisertib. (**A** and **B**) Volcano plot (**A**) and heatmap showing the 25 most differentially enriched metabolites (**B**) were generated with MetaboAnalyst after 1-way ANOVA (BH FDR, all shown *q < 0.05). See Supplemental Data Values for a list of full names. (**C**) Top 18 overrepresented pathways calculated with MetaboAnalyst based on differentially enriched metabolites are shown (BH FDR *q ≤ 0.1).

To determine how galunisertib affected blood immune cell metabolism, we performed a comprehensive hydrophilic metabolites panel, an unsupervised semiquantitative metabolomics analysis of PBMCs from 5 of the 8 macaques before and after 4 galunisertib cycles. The differential enrichment analysis (Figure 5A) revealed significantly upregulated metabolites including thiamine phosphate and nicotinamide mononucleotide (NMN) (essential for energy metabolism), histamine (important for immune responses), and deoxyguanosine and nucleotides like CTP and ATP/dGTP (critical for nucleic acid synthesis). Conversely, NAD^+^ showed significant decrease, suggesting enhanced NAD^+^-dependent enzymatic activity, which coupled with increased NMN leads to improved mitochondrial function and cellular stress response (56, 57).

The heatmap (Figure 5B) further illustrated these changes and revealed increased levels of multiple amino acids (including alanine, phenylalanine, tyrosine, and leucine), reflecting enhanced protein synthesis capacity. We also observed a pronounced decrease in polyamines (58), specifically spermidine and *N*^1^-acetylspermine, consistent with the reduced cellular proliferation detected in our scRNA-seq data above. Samples clustered distinctly by treatment condition, indicating a robust metabolic signature induced by TGF-β blockade.

Pathway analysis (Figure 5C) revealed that phenylalanine, tyrosine, and tryptophan metabolism were highly enriched, indicating substantial rewiring of aromatic amino acid processing. This was complemented by significant enrichment in pyrimidine and purine metabolism pathways, thiamine metabolism, and several other amino acid pathways. Together, these changes indicate metabolic reprogramming aligned with increased biosynthetic demands, enhanced capacity for protein synthesis, and energy generation.

**Figure 6:**
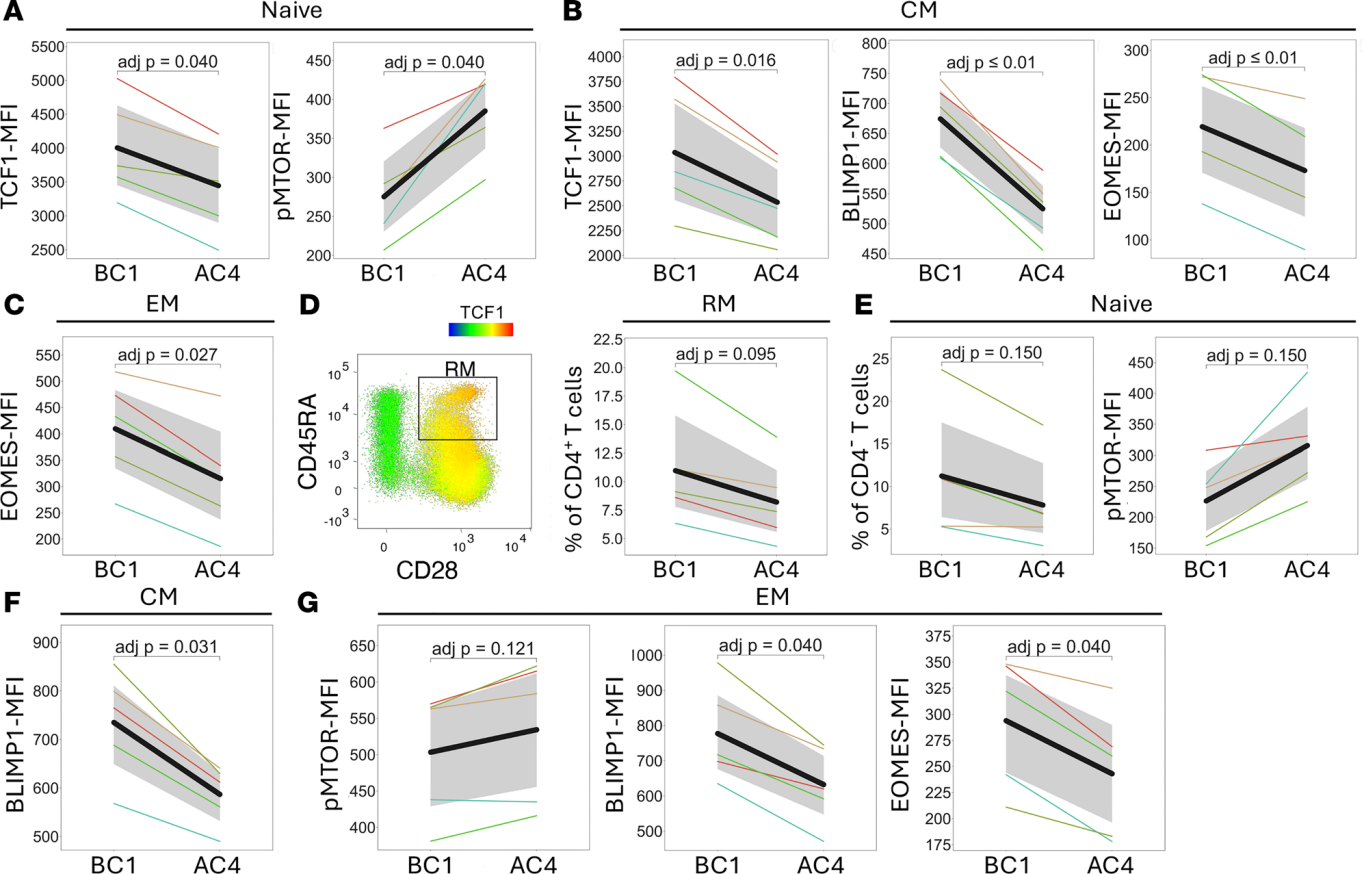
TGF-β blockade increases the levels of phospho-mTOR and decreases transcription factors involved in blood T cell differentiation. (**A**–**C** and **E**–**G**) Line plots showing significantly altered transcription factor expression (MFI) in CD4^+^ (**A**–**C**) and CD8^+^ (CD4^–^; **E**–**G**) T cell subsets (naive, central memory [CM], effector memory [EM], and resting memory [RM]) between before (BC1, before cycle 1; week 35 post-infection) and after (AC4, after cycle 4; week 49 post-infection) all 4 galunisertib cycles in PBMCs of 5 of the 8 macaques. Only variables meeting significance or near-significance thresholds (BH FDR q < 0.05) are shown. (**D**) Resting memory CD4^+^ T cells were gated as memory (CD95^+^ T cells) and then as CD45RA^+^ CD28^+^ cells. Gating strategy is shown colored by level of TCF1 expression (left), and percentage within memory CD4^+^ T cells is shown (right).

To extend our mechanistic exploration and associate this metabolic profile in PBMCs with specific changes in T cell differentiation factors, we measured levels of transcription factors and metabolic sensors with known impact on T cell phenotype and function by intracellular flow cytometry (Supplemental Table 4) before and after all 4 galunisertib cycles (BC1 vs. AC4). We found a substantial decrease in the master regulator of T cell differentiation, TCF1, across all T cell subsets (Figure 6 and Supplemental Figures 5 and 6), consistent with its higher expression in quiescent cells (59). Additionally, we observed a pronounced increase in phospho-mTOR levels in both naive CD4^+^ and CD8^+^ T cells (Figure 6, A and E), aligning with the metabolomic evidence of increased amino acid metabolism and biosynthetic activity after galunisertib. Levels of Eomes, a transcription factor important for memory T cell homeostasis, survival, and responsiveness to IL-15 (60), also decreased in both central and effector CD4^+^ and CD4^–^ (CD8^+^) T cells, suggesting exit from quiescence and enhanced effector function after galunisertib (Figure 6, B and C). This decrease at the protein level was in agreement with the transcriptional decrease detected by RNA-seq in PBMCs after galunisertib (log_2_FC = –0.65; adjusted *P* = 0.01) in data previously reported (19). Moreover, we previously described no changes in T-bet levels in CD4^+^ T cells with galunisertib treatment (19). This, together with decreased Eomes levels, translates into higher T-bet/Eomes ratio, which is typical of memory T cells exiting from quiescence after reactivation and has been found to favor differentiation over proliferation (61, 62).

Interestingly, we detected a profound decrease in Blimp1 levels in memory subsets of both CD4^+^ and CD8^+^ T cells (Figure 6, B, F, and G). While Blimp1 is normally associated with effector differentiation and TCF1 repression (63, 64), it is also known, similarly to TGF-β, to drive tissue residency (32, 65), suggesting a complex effect of TGF-β blockade potentially promoting resident T cells’ exit from mucosal tissues. Importantly, this decrease at the protein level also agreed with a decrease at the transcriptional level detected after galunisertib by bulk RNA-seq (log_2_FC = –0.51; adjusted *P* = 0.02) (19), and it is notable because of the known role of Blimp1 as an HIV transcriptional repressor in memory CD4^+^ T cells (66).

Finally, we noted a tendency toward decreased resting memory CD4^+^ T cells (expressing high levels of CD28 and TCF1; Figure 6D) and toward decreased naive CD8^+^ T cells (Figure 6E).

Collectively, these findings demonstrate that TGF-β blockade drives consistent metabolic reprogramming of CD4^+^ T cells across both blood and lymph node compartments.

### In tissues, TGF-β blockade decreases resident T cells and increases inflammatory macrophages.

**Figure 7:**
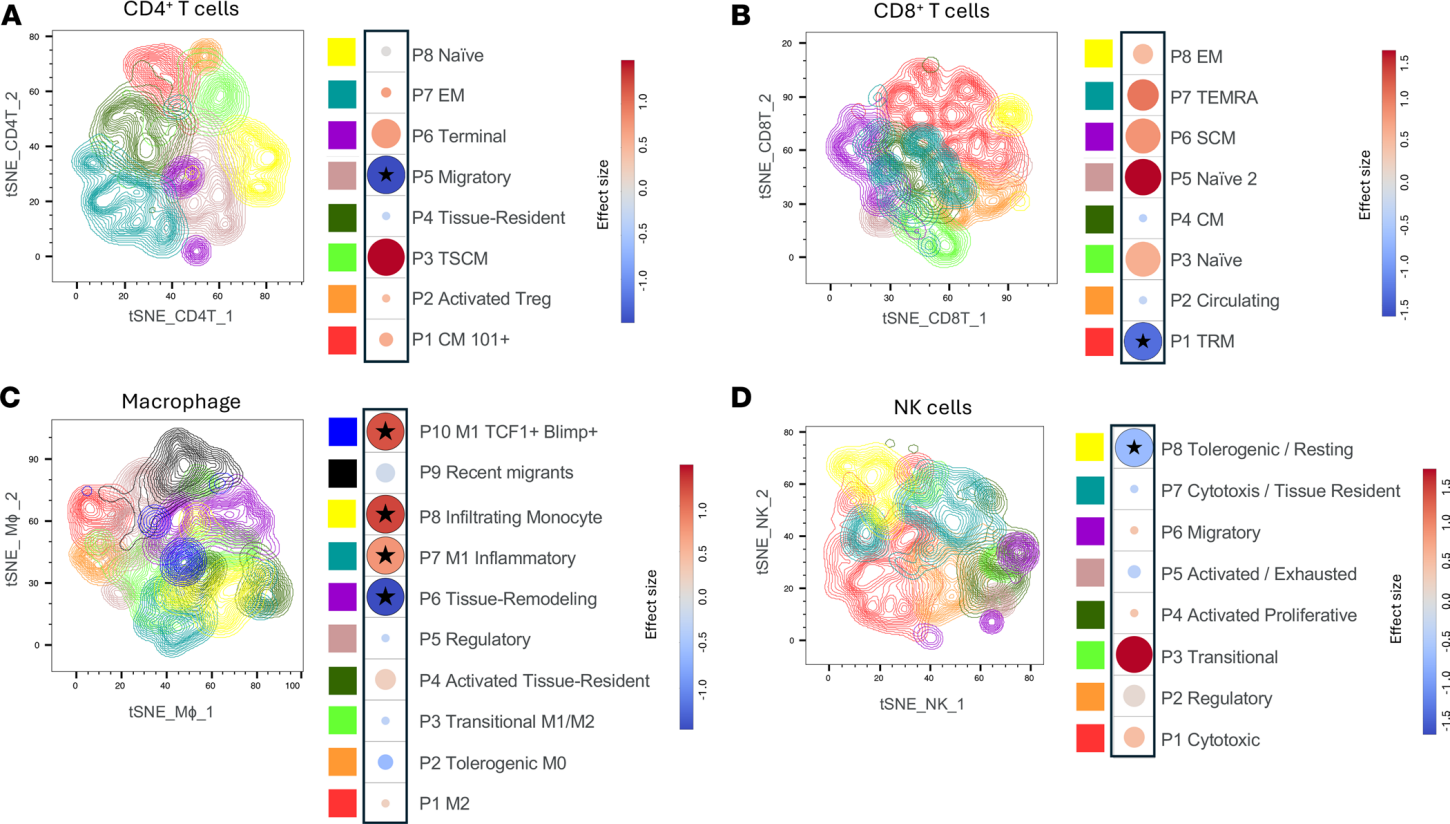
TGF-β blockade decreases resident T cells and increases inflammatory macrophages in the gut tissue. Cells from rectal biopsies were gated within live, CD45^+^, and CD4^+^ T cells (**A**), CD8^+^ T cells (**B**), macrophages (CD64^+^, **C**), and NK cells (CD3^–^, CD20^–^NKG2A^+^, **D**). Visualization of final FlowSOM clustering results mapped onto t-SNE plots is shown. Bubble plots comparing cluster proportions between before (BC1, before cycle 1; week 35 post-infection) and after (AC4, after cycle 4; week 49 post-infection) all 4 galunisertib cycles are shown next to each cluster with color proportional to the effect size of the change calculated as Hedges’ *g* (red for increased from BC1 to AC4; blue for decreased). Bubble size is proportional to the statistical significance of the change, with larger bubbles corresponding to smaller adjusted *P* values. Clusters with adjusted *q* ≤ 0.05 (Wilcoxon signed-rank test, Holm-Šidák correction) are marked with a star.

To assess the impact of TGF-β blockade on tissue immune cells, we isolated mononuclear cells from colorectal biopsies collected before and after all 4 galunisertib cycles and analyzed lymphocyte and myeloid cell subset frequencies using high-parameter spectral flow cytometry (Supplemental Table 5). After data cleaning, we gated CD4^+^ and CD8^+^ T cells from live CD45^+^ and CD3^+^ CD20^–^ populations (Supplemental Figure 7). NK cells were identified as CD20^–^CD3^–^NKG2A^+^ cells, while macrophages were defined as CD3^–^CD64^+^ cells (Supplemental Figure 7). We implemented a high-dimensional analysis strategy similar to previously reported methods (19), sequentially applying PhenoGraph (67) graph-based unsupervised clustering and FlowSOM (68) for meta-clustering, to identify cell subsets within each population. This iterative approach revealed 8 subsets within CD4^+^ and CD8^+^ T cells, 10 subsets within macrophages, and 8 within NK cells (Figure 7). Each subset was annotated based on relative expression of surface and intracellular markers (Supplemental Figure 8). Comparing subset frequencies before and after galunisertib treatment, we observed a reduction in migratory, central memory CD4^+^ T cells lacking CD69 or CD103 expression (Figure 7A and Supplemental Figure 8). In the CD8^+^ T cell compartment, we found a significant decrease in resident memory cells expressing high levels of CD69 and CD103, further suggesting that galunisertib promotes tissue exit of resident T cells (Figure 7B). Among macrophages, we identified a significant increase in all inflammatory subsets, including M1 resident and monocyte-derived macrophages (Figure 7C). Only CD25^–^ tissue remodeling/immature macrophages appeared to decrease after galunisertib. Finally, tolerogenic CCR7^+^CD69^+/–^CD103^+/–^ NK cells were significantly reduced, while transitional and cytotoxic NK cells showed a trend toward increased presence in the colorectal tissue after galunisertib (Figure 7D).

## Discussion

Our study provides compelling evidence that TGF-β blockade with galunisertib drives SIV transcription primarily through metabolic reprogramming of infected cells. By developing and applying the SCAP technique, we were able to identify and characterize SIV-infected cells actively transcribing virus in the lymph nodes during ART, revealing fundamental transcriptional and metabolic differences in virus-expressing cells before and after TGF-β blockade.

The most striking finding of our study is the dramatic reversal of metabolic quiescence in infected cells following galunisertib treatment. At baseline, SIV-RNA^+^ cells exhibited a transcriptional and metabolic profile consistent with a quiescent-like state compared with their uninfected counterparts, with downregulation of critical energy pathways including OXPHOS, glycolysis, and PI3K/AKT/mTOR signaling. This aligns with the emerging understanding that viral latency is linked to metabolic dormancy (69), where reduced energy production creates an environment unfavorable for viral transcription (22, 70, 71). It expands this concept, suggesting that metabolic dormancy is also present in infected cells actively transcribing virus during ART, with the caveat that we do not know whether any of the transcribed virus either before or after galunisertib was replication competent. Notably, these findings align with the only other published analysis of HIV transcript–expressing CD4^+^ T cells in ART-suppressed individuals, which similarly found upregulation of genes associated with inhibition of activation and cellular quiescence in comparison with transcript-negative cells (34).

The metabolic shift driven by galunisertib was similar in both virus-positive and -negative cells, and it was comprehensive, affecting multiple interconnected pathways. The concurrent upregulation of glycolysis and fatty acid metabolism with downregulation of OXPHOS and WNT/β-catenin signaling suggests a classical metabolic shift from low to high energy demand (72), while increased amino acid metabolism supports protein synthesis leading to increased biosynthetic activities in virus-expressing cells after galunisertib treatment (73). The downregulation of OXPHOS coupled with upregulation of OXPHOS genes (particularly those involved in mitochondrial biosynthesis) suggests a compensatory adaptation to higher energy demands rather than direct enhancement of mitochondrial respiration. This pattern is consistent with metabolically active effector T cells (74), which primarily rely on glycolysis while maintaining sufficient mitochondrial capacity to sustain their increased energetic needs (75). Supporting this, via metabolomics analysis of PBMCs, we observed decreased NAD^+^ coupled with increased NMN, indicating enhanced NAD^+^-dependent enzymatic activity that sustains mitochondrial function (76).

Importantly, this metabolic rewiring occurred without inducing T cell proliferation, as evidenced by the downregulation of the centrosomal protein CEP44 (77) and the decreased polyamine levels detected in our metabolomic analysis (78). This supports our previous observation that TGF-β blockade specifically uncouples cellular activation from proliferation (19), potentially offering a safer approach to latency reversal compared with strategies that induce widespread T cell activation and expansion (79, 80).

The metabolic requirements for HIV/SIV transcription and replication include increased glucose uptake, enhanced glycolysis, elevated biosynthetic processes, and upregulated glutaminolysis (21, 22, 81). Our findings indicate that TGF-β blockade satisfies these requirements by promoting an “effector-like” metabolic state in latently infected cells. This metabolic shift occurs alongside the specific upregulation of *JUN* and concomitant downregulation of *Blimp1*, which likely directly enhance viral transcription through binding of the AP-1 complex to the viral long-terminal repeat (*JUN*) (82) and reduced transcriptional inhibition (*Blimp1*) (66).

Beyond CD4^+^ T cells, our high-dimensional flow cytometry analyses revealed that TGF-β blockade orchestrates a coordinated immunological shift across multiple cell types in tissues. The decrease in resident memory T cells expressing high levels of CD69 and CD103 is supported by the finding of decreased Blimp1 expression in T cells in circulation. This exit of T cells from the mucosa coupled with the increase in inflammatory macrophages suggests a reshaping of the tissue immune environment. The widespread effect of galunisertib and its impact in tissues may contribute to its efficacy in reversing latency in vivo, as it targets not only CD4^+^ T cells but also other myeloid cells and possibly leads to the recirculation of reservoir-rich tissue-resident cells (83, 84).

Our findings have significant implications for HIV cure strategies. By demonstrating that metabolic reprogramming may be sufficient to reverse viral latency without inducing proliferation, we highlight a potential advantage of TGF-β blockade — and other metabolism-focused strategies — over traditional latency-reversing agents that rely on T cell activation but also expand these cells. The simultaneous enhancement of cellular metabolism and stress responses provides a mechanistic explanation for why this approach effectively reactivates virus while potentially limiting cytopathic effects and systemic inflammation (19).

Furthermore, our results suggest that the efficacy of latency-reversing strategies may depend on their ability to also fulfill the specific metabolic requirements of viral transcription (85). This is particularly relevant considering the heterogeneity of viral reservoirs across different tissue compartments and cell types, each with distinct metabolic profiles.

By integrating our transcript capture assay with Seahorse metabolic profiling and metabolomics analyses, we provide comprehensive evidence that TGF-β inhibition promotes an effector-like metabolic state, characterized by increased glycolytic activity, enhanced mitochondrial biosynthesis to meet elevated energetic demands, and reduced spare respiratory capacity typical of effector T cells.

The limitations of our study include the relatively small sample size (8 macaques analyzed before and after treatment) and the lack of a control, untreated group of macaque samples. The former issue also limited the number of on-ART SIV-RNA^+^ T cells analyzed, leading to a very small sample size (*n* = 31) before intervention, which was underpowered to detect significant DEGs in comparison with uninfected/transcriptionally silent T cells. Hence, we focused on highly significant uncorrected DEGs that, together with GSEA, suggested that on-ART viral transcription is associated with a quiescent/exhausted phenotype. A similar limitation was present in the post-galunisertib analysis in which we compared 38 vRNA^+^ cells to thousands of vRNA^–^ cells. Finally, because SCAP specifically enriches for cells actively transcribing viral RNA, it does not capture the large fraction of the reservoir that remains transcriptionally silent during ART. Thus, our analysis describes the metabolic features of the transcriptionally active reservoir, while cells with silent proviruses may differ substantially in metabolic state. Regarding the lack of a control group, this was tempered by the fact that the macaques were on ART for over 8 months before treatment and acting as their own controls. Finally, the technical limitations include that cells transcribing the virus could only be analyzed transcriptionally, while the bulk of the CD4^+^ T cells in the lymph nodes and blood could be assayed also by Seahorse and metabolomics.

The SCAP technique developed for this study represents a significant methodological advance, enabling the sensitive identification and characterization of SIV-RNA^+^ cells within scRNA-seq data. This approach captures cells with varying levels of viral transcription, not just those with high expression detected by standard scRNA-seq at high sequencing depth. The ability to detect transcriptionally active virus in suppressed animals provides a powerful tool for future studies investigating reservoir dynamics and response to interventions and can be easily adapted to HIV and tissues from PLWH on treatment.

In conclusion, our study establishes a mechanistic link between TGF-β signaling, cellular metabolism, and viral latency. Our data suggest that TGF-β signaling serves as a key mediator in maintaining viral latency through metabolic suppression. By demonstrating that TGF-β blockade drives SIV transcription through enhanced cellular metabolism, we highlight a — to our knowledge — novel pathway for latency reversal that differs from conventional approaches focused on direct transcriptional activation. This metabolic perspective on latency reversal may inform more effective therapeutic strategies that combine metabolic modulation with immune enhancement to achieve sustained viral clearance, bringing us closer to the goal of HIV remission or cure.

## Methods

### Sex as a biological variable.

Our study exclusively examined female macaques due to constraints in nonhuman primate availability at the time of study initiation. Whether the findings are relevant to male macaques and humans will require additional studies.

### Study design.

A total of 8 adult female Indian-origin rhesus macaques (*Macaca mulatta*; Mamu A*01, B*08, and B*17 negative) were used for the study described (19) (Figure 1A). Rhesus macaques were infected with 300 TCID_50_ (50% tissue culture infectious dose) of the barcoded SIVmac239M2 stock intravenously, and ART (tenofovir [PMPA] at 20 mg/mL, emtricitabine [FTC] at 40 mg/mL, and dolutegravir [DTG] at 2.5 mg/mL) was initiated at week 6 after infection. Galunisertib treatment was initiated at week 35 after infection. Galunisertib powder (MedChemExpress) was dissolved in water and given orally in a treat twice daily at 20 mg/kg. Four cycles of 2 weeks of daily treatment with a 2-week washout period were performed. Additional details about this study, including plasma and tissue viral loads and immunoPET/CT evaluation of the viral reservoir in tissues, can be found in ref. 19.

### Single-cell RNA sequencing.

Lymph node cells isolated by passing of the cleaned tissue through a 70 μm strainer were thawed and chemically fixed using Evercode Fixation kit (Parse Biosciences). cDNA synthesis and library preparation were performed using Evercode v3 chemistry. Libraries were validated by capillary electrophoresis on a Fragment Analyzer (Agilent) and sequenced using one S4 flow cell of the Illumina NovaSeq 6000 yielding greater than 20,000 reads per cell. Raw sequencing data were demultiplexed, aligned to Mmul10, and subjected to quality control (QC) with the SplitPipe data processing pipeline (v1.2.1, Parse Biosciences) with default settings.

scRNA-seq whole-transcriptome data were processed with Seurat (v5.2.1) in R, starting with QC filtering to remove low-quality cells and doublets. Cells were retained if they had between 50 and 8,000 detected genes and total unique molecular identifier (UMI) counts between 450 and 200,000, while those with mitochondrial gene content exceeding 25% were excluded. These filters were chosen as optimal to retain a good amount of SIV transcript–positive cells (see SCAP below), while excluding false-positive and low-quality cells. Normalization was performed using the deconvolution-based size factor method from the scran package (v1.34.0; Bioconductor), followed by log-transformation. Dimensionality reduction was first performed using principal component analysis on variable features. Batch-aware integration across samples was performed using the Harmony algorithm on the top 15 principal components. Subsequently, UMAP was computed on the Harmony-corrected embeddings using the Euclidean distance metric. Clustering was then performed on the Harmony-reduced space using the Louvain algorithm testing resolutions from 0.05 to 1.0, with a final resolution of 0.2 selected for downstream analysis. Differential expression analysis was performed using a paired statistical design (before compared with after galunisertib treatment) with the hurdle model from the model-based analysis of single-cell transcriptomics (MAST) framework (Bioconductor), as implemented in the FindMarkers function in Seurat (v5.2.1). A latent variable was incorporated to account for sample-level variation. After filtering to retain only protein-coding genes, annotations were assigned using the Ensembl human gene database via the biomaRt package (v2.62.1). Gene set enrichment analysis (GSEA) was performed on all protein-coding log_2_ fold change–ranked genes using the fgsea package (v1.32.2). Hallmark gene sets were sourced from the Molecular Signatures Database (MSigDB), while KEGG gene sets were obtained from the C2 CP: KEGG subcategory of MSigDB for *Homo sapiens* using the msigdbr package (v7.5.1), and a subset of 66 metabolism-associated pathways was manually selected for focused enrichment analysis (Supplemental Table 2). To assess the quiescent state of CD4^+^ T cells, a quiescence score was computed using the AddModuleScore function in Seurat, based on a manually curated gene set provided in Supplemental Table 3. This scoring approach enabled quantification of quiescence-associated transcriptional programs at the single-cell level.

### SIV Transcripts Capture Assay based on the Parse Biosciences analysis.

To sensitively identify SIV-infected cells expressing viral transcripts at single-cell resolution, we developed a custom enrichment strategy termed the SIV Transcripts Capture Assay with Parse Biosciences (SCAP), as illustrated in Supplemental Figure 2A. This method uses the previously prepared full whole-transcriptome single-cell library, followed by the custom Parse Gene Select including a blocker to minimize nonspecific binding and incubation with 314 (120 bp) biotinylated probes targeting the SIV coding regions (Supplemental Data 1). The probes were custom-designed by Twist Bioscience to span all possible SIV transcripts and be highly specific, cover each base at least 4 times, and avoid crossing exon boundaries whenever possible. SIV-enriched transcripts were isolated through magnetic capture and subjected to PCR amplification, generating an SIV-enriched library that was sequenced with 300 million reads.

SCAP sequences, as well as sequences from the uncaptured libraries (to determine uncaptured coverage; Supplemental Figure 2B), were initially mapped against SIVmac239 reference genome (M33262.1) using Burrows-Wheeler Aligner (BWA) software (PMID: 19451168) with the BWA-MEM algorithm. Subsequently, SAMtools (PMID: 19505943) was used for SAM-to-BAM conversion and sorting, as well as to estimate the depth of coverage of SIV sequences. After confirming successful capture of SIV sequences with the SCAP approach (and minimal SIV presence in the uncaptured library), we performed the PARSE-specific data analysis using the SplitPipe data processing pipeline (v1.2.1) changing several of the default filters (--tscp_min 1; --post_min_map_frac 0.005; --dge_min_start_tscp 100) to map the reads against the SIVmac239 reference genome to quantify the different SIV transcripts transcribed per cell. This analysis yielded a total of 706 putative SIV transcript–positive cells, but none in the SIV-uninfected control sample. Afterward, SIV reads were integrated with the whole-transcriptome quantification data and associated metadata using the cell-specific barcodes present in the captured SIV sequences. A total of 552 of the 706 SIV-RNA^+^ cells identified by SCAP were also found in the whole-transcriptome library processed with standard SplitPipe data processing pipeline as described above. The 552 SIV-RNA^+^ cells were funneled in the whole-transcriptome QC pipeline described above where cells were retained if they had between 50 and 8,000 genes, total UMI counts between 450 and 200,000, and <25% mitochondrial gene content (*n* = 127 cells remaining).

### Seahorse metabolic profiling of CD4^+^ T cells from lymph nodes.

CD4^+^ T cells were sorted from lymph node cells from 5 of the 8 remaining macaques on a BD FACSymphony S6 sorter as live, CD45^+^CD3^+^CD8^–^ cells and resuspended in Agilent Seahorse XF RPMI medium supplemented with 10 mM glucose, 1 mM pyruvate, and 2 mM glutamine. Ninety thousand cells were seeded per well of an HS Mini Seahorse plate in duplicate or triplicate and subjected to sequential injections of oligomycin (1.5 μM), BAM15 (2.5 μM), and rotenone/antimycin A (0.5 μM). The HS Mini plate and sensor cartridge were loaded into an Agilent HS Mini Analyzer and run using the manufacturer’s T Cell Metabolic Profiling protocol. The data were analyzed using Agilent Seahorse Analytics software.

### PBMC comprehensive hydrophilic metabolites panel.

PBMCs from 5 of the 8 macaques for which time points immediately before (week 35, BC1) and after (week 49, AC4) the 4 galunisertib cycles were available were thawed and rinsed with 0.9% NaCl and lysate in 80% methanol at –80°C. Lysate was incubated overnight at –20°C, vortexed, and centrifuged at 20,000*g* for 15 minutes. Supernatant was removed and stored at –80°C until liquid chromatography/mass spectrometry analysis at the Northwestern University metabolomics core. The peak area data were normalized by cell number and total ion count and log-transformed. Data were further analyzed using MetaboAnalyst, a web-based metabolomics analysis platform developed by the Xia Lab, and 1-way ANOVA to generate the volcano plot and heatmap to visualize the top 25 different metabolites. The data were further processed for pathway enrichment analysis.

### Analysis of transcription factors in PBMCs.

PBMCs from 5 of the 8 macaques for which time points immediately before (week 35, BC1) and after (week 49, AC4) the 4 galunisertib cycles were available were thawed and stained with a panel of antibodies against transcription factors (Supplemental Table 4). After extracellular staining, fixation, and permeabilization, the intracellular antibodies were incubated in permeabilization buffer from the eBioscience Foxp3/Transcription Factor Staining Set after mouse IgG incubation. After 45 minutes of incubation at room temperature, the antibodies were washed and cells acquired immediately using a 4L Cytek Aurora. Data cleanup and subset gating were performed manually in FlowJo v10 (BD Biosciences). The geometric mean intensity of fluorescence for each of the transcription factors was compared between time points as detailed in *Statistics* below.

### Analysis of rectal biopsy cells.

Mononuclear cells were isolated by enzymatic digestion from rectal biopsies collected from the 8 macaques before cycle 1 (BC1, week 35 post-infection) and after cycle 4 of galunisertib treatment (AC4, week 49 post-infection), thawed in the presence of 50 U/mL of endonuclease in RPMI, and filtered with a 70 μm strainer. Extracellular and intracellular staining with the antibodies listed in Supplemental Table 5 was performed using the eBioscience Foxp3/Transcription Factor Staining Buffer Set after mouse IgG incubation to prevent nonspecific binding. Data were acquired in Cytek Aurora 4L, unmixed with autofluorescence removal, and compensated in SpectroFlo software (Cytek). Data were analyzed in FlowJo v10 by first gating on single, live, and CD45^+^ cells. CD3^+^ cells were gated as either CD4^+^ or CD8^+^ T cells. Within the CD3^–^ population, macrophages and NK cells were identified using CD30, CD64, and NKG2A (Supplemental Figure 7). After downsampling with the DownSample v3 plug-in in FlowJo, samples within each cell type were concatenated and processed separately for dimensionality reduction. T-distributed stochastic neighbor embedding (t-SNE) was performed to map and display the final clustering results on a 2-dimensional space. To ensure interpretability, surface markers used in the gating or those lacking detectable signal within each cell type were excluded from analysis. PhenoGraph (66) was first used to determine the optimal number of clusters as input for FlowSOM (68). The Taylor score from Euclide, a FlowJo plug-in, was used to evaluate clustering quality and determine the most suitable clustering strategy for our dataset. Finally, the candidate clusters were manually evaluated based on known immunophenotypic features to determine the final cluster annotation.

### Declaration of generative AI.

In the preparation of the manuscript, the authors used Claude 3.7 Sonnet to improve readability of the text. After using this tool, the authors reviewed and edited the content, as necessary. The authors take full responsibility for the content of the publication.

### Statistics.

GraphPad Prism v10, R, and Python were used for statistical analysis and data visualization.

For the analysis of levels of transcription factors, the data at each time point were tested for normality using the Shapiro-Wilk test followed by 2-tailed paired *t* tests for normally distributed data or Wilcoxon matched-pairs test for non-normally distributed data. *P* values were adjusted for multiple testing using the Benjamini-Hochberg false discovery rate (BH FDR). A negative binomial mixed-effects model was implemented to assess differences in cell proportions across clusters in the CD4^+^ T cell transcriptome analysis (Figure 3, D and E) by subset with zero inflation for the SIV-RNA^+^ cells, and *P* values were adjusted for multiple testing using BH FDR (response variable: cell count; predictor: condition). To assess the differences in the relative frequency of each cluster in the rectal biopsies analysis (Figure 7), the Wilcoxon signed-rank test function in the Pingouin statistical package was run in Python (version 3.10). *P* values were adjusted for multiple testing using the BH FDR (Figure 3, D and E) and the Holm-Šidák correction (Figure 7). Statistical comparison of quiescence module scores between SIV^+^ cells before (BC1) and after (AC1) galunisertib was performed using the Wilcoxon signed-rank test. Significance was defined as *P* ≤ 0.05 (Prism v10).

### Study approval.

All animal experiments were conducted following guidelines established by the Animal Welfare Act and the NIH for housing and care of laboratory animals and performed in accordance with institutional regulations after review and approval by the Institutional Animal Care and Use Committee of the University of Louisiana at Lafayette (2021-8821-002; protocol 8821-01).

### Data availability.

All relevant data are included in the article or supplemental material. Values for all data points in graphs are reported in the Supporting Data Values file. Raw data files are available to be shared upon request to corresponding author. All RNA sequencing data originating from this study were deposited in the NCBI’s Gene Expression Omnibus database (GEO GSE244871).

## Author contributions

RA and JHG performed data analysis and wrote the manuscript. JK, CMW, KLY, CAS, and AMK contributed to sample processing and assays. JTP contributed to data analysis. MA, DB, and FJV coordinated sample collection from the nonhuman primate study. RLR analyzed SCAP data and contributed to statistical analysis. EM, JA, and CC conceptualized the studies, analyzed the data, and wrote the manuscript.

## Funding support

This work is the result of National Institutes of Health (NIH) funding, in whole or in part, and is subject to the NIH Public Access Policy. Through acceptance of this federal funding, the NIH has been given a right to make the work publicly available in PubMed Central.

- Intramural Research Program of the NIH
- NIH grant R01 AI176599 to EM.
- Resource for Nonhuman Primate Immune Reagents (R24 OD010947) to FJV.
- The Lurie Cancer Center is supported in part by a National Cancer Institute Cancer Center Support Grant (P30 CA060553)

## Supplementary Material

Supplemental data

Supplemental data set 1

Supporting data values

## Version 1 02/23/2026

Electronic publication
