# Supplementary material for: Single-cell capture of on-ART SIV transcription reveals TGF-β–mediated metabolic control of viral latency
Source: JCI Insight. 2026 Feb 23;11(4):e198810. doi: 10.1172/jci.insight.198810 (PMC12956001; doi:10.1172/jci.insight.198810)
Supplement: Supplemental data [file jciinsight-11-198810-s216.pdf]

Supplementary Figures

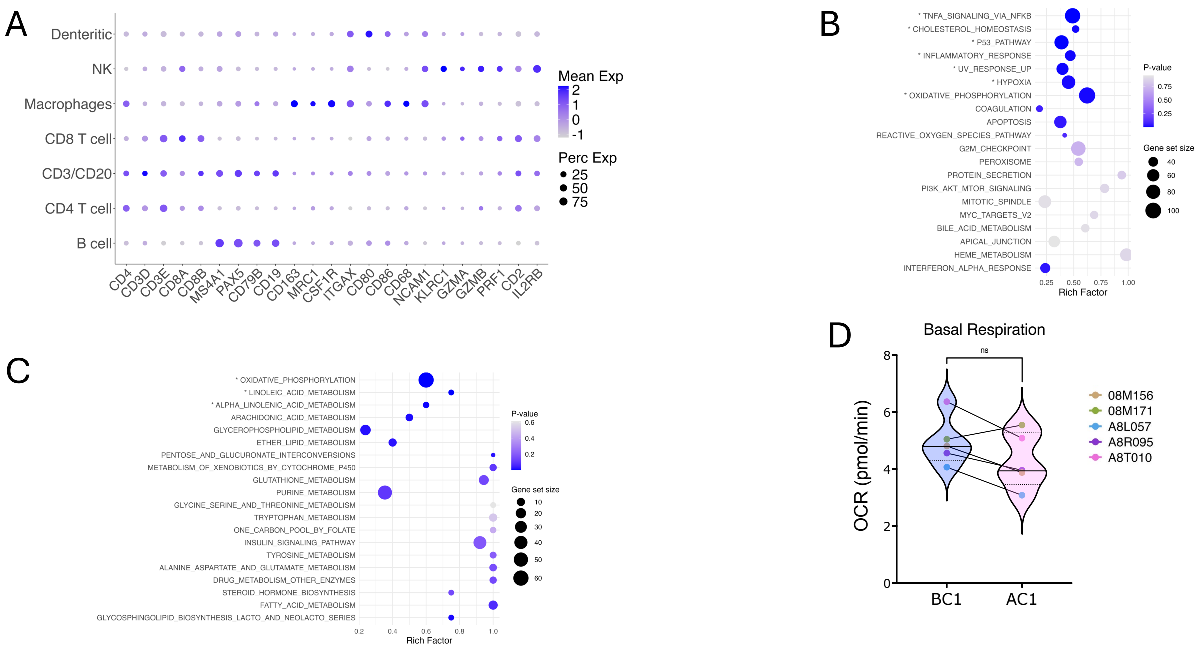

Figure S1. Gene expression data used for annotation of lymph node clusters and basal respiration before and after galunisertib. A) Bubble plot showing expression (mean normalized and Z-scaled counts proportional to the color; size proportional to the percentage of cells) of markers used for canonical annotation of major immune cell subsets. B-C) Bubble plot version of GSEA analysis data for Hallmark (B) and metabolic Kegg (C) sets indicating the gene set size (size of the bubble) and the p-value (intensity of color) from Figure 1. D)Basal oxygen consumption rates of sorted CD4^{+} T cells before (BC1) compared to after (AC1) the first 2-weeks cycle with galunisertib for 5 macaques with enough stored cells for sorting as measured by Seahorse.

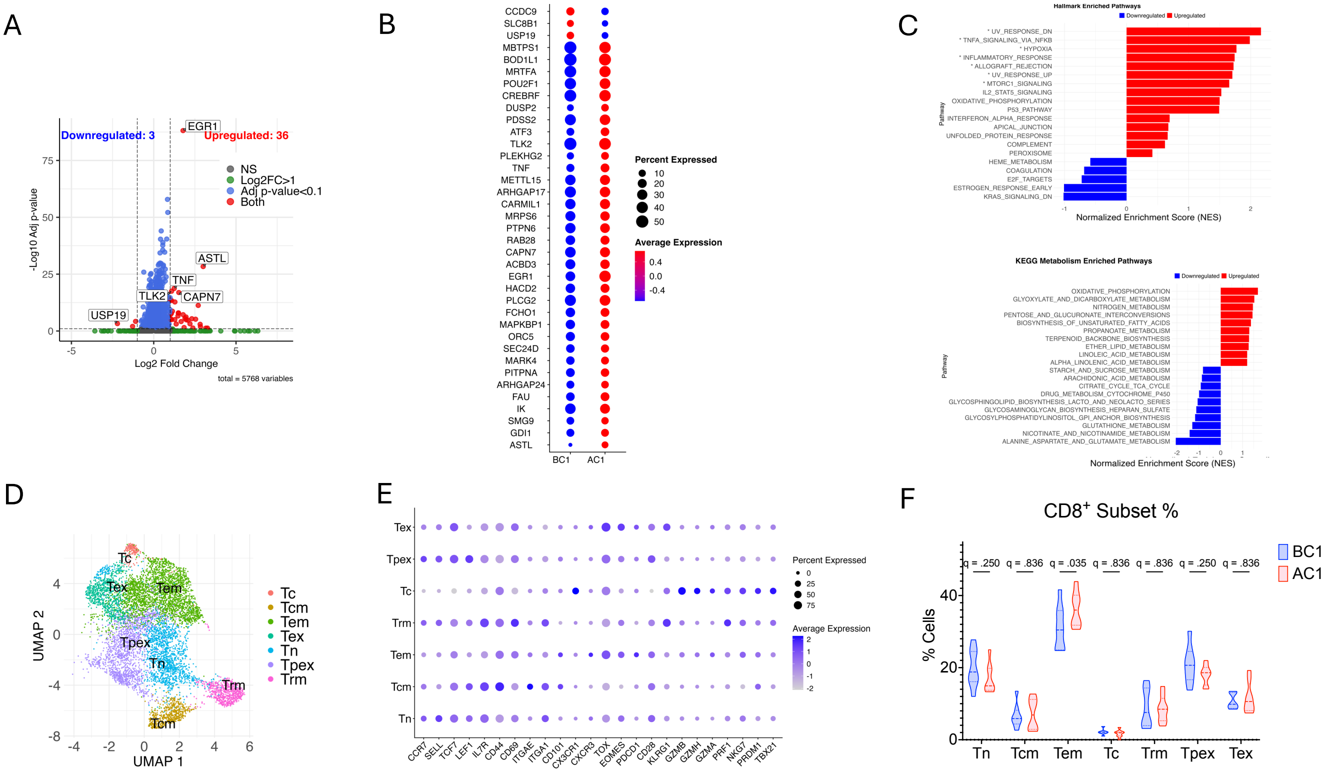

Figure S2 Galunisertib upregulates inflammatory responses in lymph node $CD8^+$ T cells. A) Volcano plot showing differentially expressed genes (DEGs) from the comparison of lymph nodes $CD8^+$ T cells before and after the first 2 weeks of galunisertib (cycle 1; BC1 vs AC1) by MAST hurdle model. B) Bubble plot showing significant DEGs (B-H FDR adjusted $*q \leq 0.05$ and log2FC ≥1). C) Enriched Hallmark and KEGG metabolic pathways in total $CD8^+$ T cells based on GSEA (BC1 vs. AC1). Upregulated pathways (red) and downregulated pathways (blue) are shown with their respective NES (B-H FDR $*q \leq 0.1$). D) UMAP projection of scRNA-seq data from lymph node tissue, showing cluster annotation of distinct $CD8^+$ T cell subtypes based on E). E) Bubble plot displaying the average normalized expression of selected markers used for $CD8^+$ T cell annotation (mean normalized and Z-scaled counts proportional to the color; size proportional to the percentage of cells). F) The frequencies of $CD8^+$ T cell subsets before (BC1) and after (AC1) galunisertib are shown compared by mixed effect models and BH FDR correction.

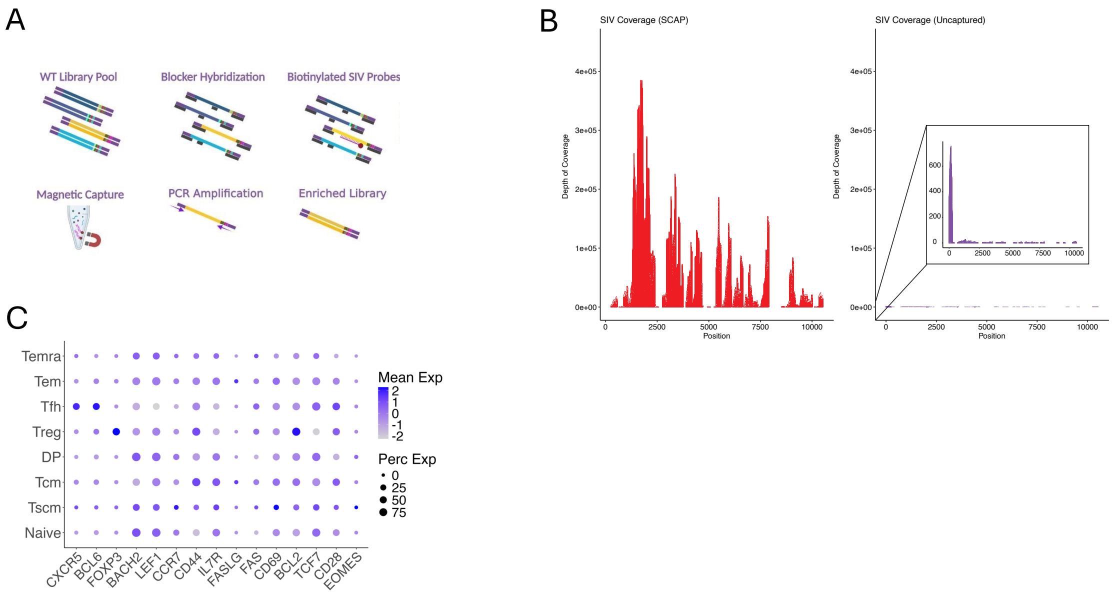

Figure S3. SCAP workflow and SIV capture. A) The workflow of SCAP using Parse Biosciences technology gene select kit. Blocker hybridization decreased non-specific probe binding, while 314 biotinylated SIV probes selectively captured viral RNA. Magnetic beads isolated these sequences, followed by PCR amplification and sequencing. B) Depth of coverage of SIV-mapped sequences resulted from SCAP (left) and the uncaptured whole transcriptome library for all samples. Reds obtained for each experiment were aligned to SIVmac239 reference genome and the depth of coverage was quantified as the number of reads mapped per position. C) Bubble plot displaying the average normalized expression of selected markers used for annotation of $CD4^+$ T cell clusters is shown (mean normalized and Z-scaled counts proportional to the color; size proportional to the percentage of cells).

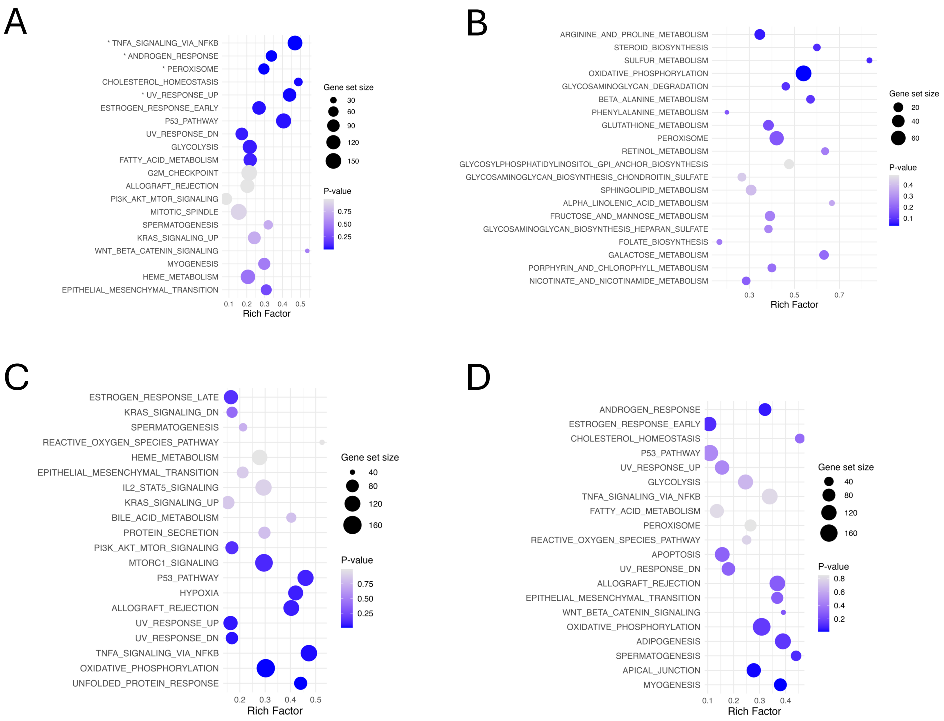

Figure S4. Bubble plot versions of the GSEA analysis shown in Figures 3 and 4 with indicated gene set size and p-values.

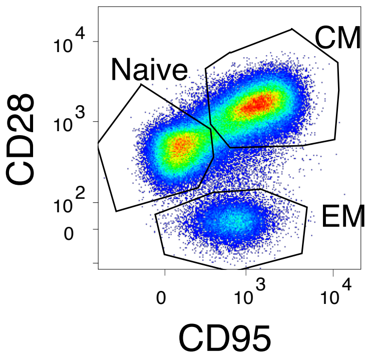

Figure S5. Gating strategy of CD4 T cell subsets used for comparisons of transcription factors. Live lymphocytes were gated within $CD3^+$ $CD4^+$ cells and then as above for Figure 6 and Figure S6 data.

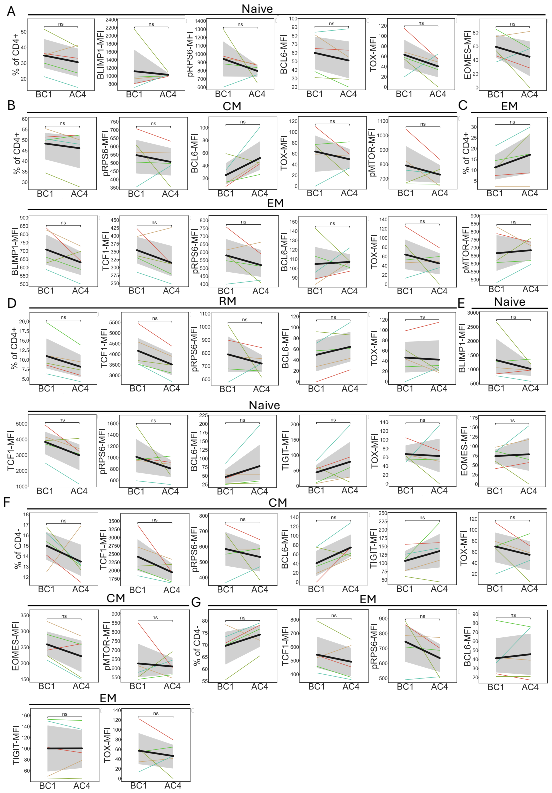

Figure S6 Transcription factors not altered in blood T cells by galunisertib treatment. Line plots showing non significantly (BH-FDRq>0.05) altered transcription factors expression (MFI) in CD4^{+} (A-D) and CD8^{+} (CD4^{-}; E-G) T cell subsets before (BC1) galunisertib compared to after the four galunisertib cycles (AC4= after cycle 4). CM= central memory, EM= effector memory, and RM= resting memory.

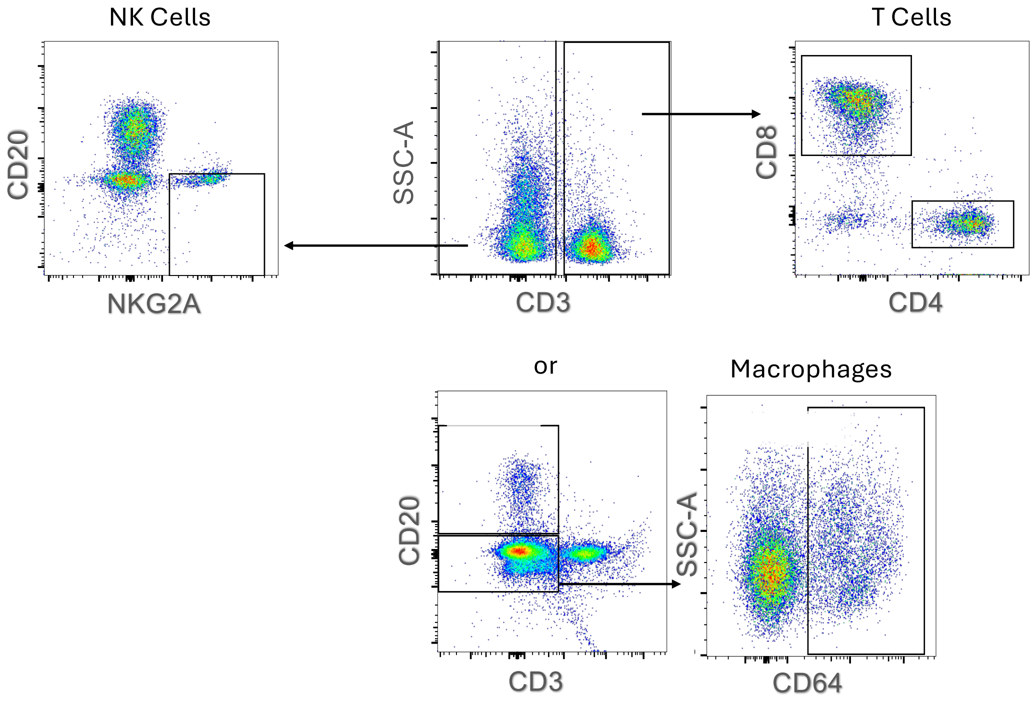

Figure S7 Gating strategy for colorectal tissue cells. Live mononuclear cells isolated from colorectal biopsies before and after 4 cycles of galunisertib were gated as shown before each subset was analyzed with the high dimensional pipeline described in the methods.

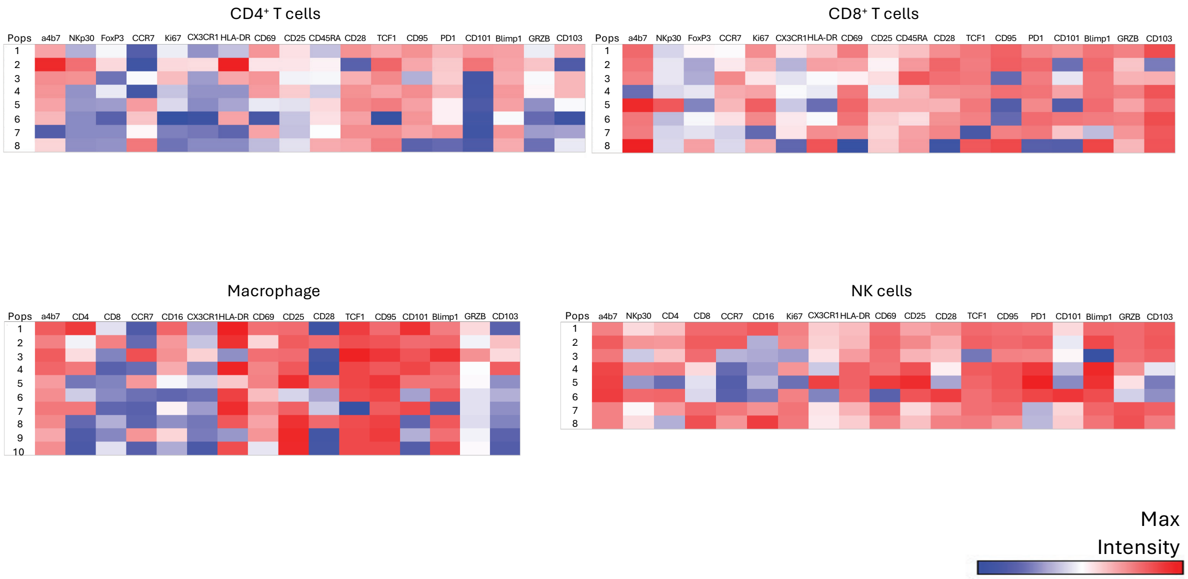

Figure S8 Heatmaps of rectal biopsy cell populations generated by FlowSOM. FlowSOM was run with Phenograph's suggested numbers of clusters on cleaned, live, singlet populations gated as shown in Figure S5 from BC1 and AC4 data (before compared to after all four galunisertib cycles). Heatmap of Z-scaled MFI for each marker in each FlowSOM population is shown.

Table S1 List of Metabolism-Associated Pathways Obtained from the C2 CP: KEGG Subcategory

1.	KEGG_ALANINE_ASPARTATE_AND_GLUTAMATE_METABOLISM
2.	KEGG_ALPHA_LINOLENIC_ACID_METABOLISM
3.	KEGG_AMINO_SUGAR_AND_NUCLEOTIDE_SUGAR_METABOLISM
4.	KEGG_ARACHIDONIC_ACID_METABOLISM
5.	KEGG_ARGININE_AND_PROLINE_METABOLISM
6.	KEGG_ASCORBATE_AND_ALDARATE_METABOLISM
7.	KEGG_BETA_ALANINE_METABOLISM
8.	KEGG_BIOSYNTHESIS_OF_UNSATURATED_FATTY_ACIDS
9.	KEGG_BUTANOATE_METABOLISM
10.	KEGG_CITRATE_CYCLE_TCA_CYCLE
11.	KEGG_CYSTEINE_AND_METHIONINE_METABOLISM
12.	KEGG_DRUG_METABOLISM_CYTOCHROME_P450
13.	KEGG_DRUG_METABOLISM_OTHER_ENZYMES
14.	KEGG_ETHER_LIPID_METABOLISM
15.	KEGG_FATTY_ACID_METABOLISM
16.	KEGG_FOLATE_BIOSYNTHESIS
17.	KEGG_FRUCTOSE_AND_MANNOSE_METABOLISM
18.	KEGG_GALACTOSE_METABOLISM
19.	KEGG_GLUTATHIONE_METABOLISM
20.	KEGG_GLYCEROLIPID_METABOLISM
21.	KEGG_GLYCEROPHOSPHOLIPID_METABOLISM
22.	KEGG_GLYCINE_SERINE_AND_THREONINE_METABOLISM
23.	KEGG_GLYCOLYSIS_GLUCONEOGENESIS
24.	KEGG_GLYCOSAMINOGLYCAN_BIOSYNTHESIS_CHONDROITIN_SULFATE
25.	KEGG_GLYCOSAMINOGLYCAN_BIOSYNTHESIS_HEPARAN_SULFATE
26.	KEGG_GLYCOSAMINOGLYCAN_BIOSYNTHESIS_KERATAN_SULFATE
27.	KEGG_GLYCOSAMINOGLYCAN_DEGRADATION
28.	KEGG_GLYCOSPHINGOLIPID_BIOSYNTHESIS_GANGLIO_SERIES
29.	KEGG_GLYCOSPHINGOLIPID_BIOSYNTHESIS_GLOBO_SERIES
30.	KEGG_GLYCOSPHINGOLIPID_BIOSYNTHESIS_LACTO_AND_NEOLACTO_SERIES
31.	KEGG_GLYCOSYLPHOSPHATIDYLINOSITOL_GPI_ANCHOR_BIOSYNTHESIS
32.	KEGG_GLYOXYLATE_AND_DICARBOXYLATE_METABOLISM
33.	KEGG_INOSITOL_PHOSPHATE_METABOLISM
34.	KEGG_INSULIN_SIGNALING_PATHWAY
35.	KEGG_LIMONENE_AND_PINENE_DEGRADATION
36.	KEGG_LINOLEIC_ACID_METABOLISM
37.	KEGG_LYSINE_DEGRADATION
38.	KEGG_METABOLISM_OF_XENOBIOTICS_BY_CYTOCHROME_P450
39.	KEGG_NICOTINATE_AND_NICOTINAMIDE_METABOLISM
40.	KEGG_NITROGEN_METABOLISM
41.	KEGG_ONE_CARBON_POOL_BY_FOLATE
42.	KEGG_OXIDATIVE_PHOSPHORYLATION
43.	KEGG_PANTOTHENATE_AND_COA_BIOSYNTHESIS
44.	KEGG_PENTOSE_AND_GLUCURONATE_INTERCONVERSIONS
45.	KEGG_PENTOSE_PHOSPHATE_PATHWAY
46.	KEGG_PEROXISOME
47.	KEGG_PHENYLALANINE_METABOLISM
48.	KEGG_PORPHYRIN_AND_CHLOROPHYLL_METABOLISM
49.	KEGG_PROPANOATE_METABOLISM
50.	KEGG_PURINE_METABOLISM
51.	KEGG_PYRIMIDINE_METABOLISM
52.	KEGG_PYRUVATE_METABOLISM
53.	KEGG_RETINOL_METABOLISM
54.	KEGG_RIBOFLAVIN_METABOLISM
55.	KEGG_SELENOAMINO_ACID_METABOLISM
56.	KEGG_SPHINGOLIPID_METABOLISM
57.	KEGG_STARCH_AND_SUCROSE_METABOLISM
58.	KEGG_STEROID_BIOSYNTHESIS

59.	KEGG_STEROID_HORMONE_BIOSYNTHESIS
60.	KEGG_SULFUR_METABOLISM
61.	KEGG_TAURINE_AND_HYPOTAURINE_METABOLISM
62.	KEGG_TERPENOID_BACKBONE_BIOSYNTHESIS
63.	KEGG_TRYPTOPHAN_METABOLISM
64.	KEGG_TYROSINE_METABOLISM
65.	KEGG_VALINE_LEUCINE_AND_ISOLEUCINE_BIOSYNTHESIS
66.	KEGG_VALINE_LEUCINE_AND_ISOLEUCINE_DEGRADATION

Table S2 List of Genes Involved in Mitochondrial Biosynthesis and Modulation

**Mitochondrial biogenesis regulators:**	
PPARGC1A (PGC1α)	Master regulator of mitochondrial expansion
PPARGC1B (PGC1β)	Supports biogenesis
NRF1	Drives expression of respiratory genes
NFE2L2	Drives expression of respiratory genes
GABPA	Coordinates respiratory chain expression
TFAM	Increases mtDNA replication and transcription
ESRRA	Works with PGC1α to increase mitochondrial ge[illegible]
**Mitochondrial dynamics**	
OPA1	Promotes fusion
DNM1L	Decreases fission
FIS1	Decreases fission
**Quality control**	
PINK1	Monitor mitochondrial health
PRKN	Maintain quality
BNIP3	Increases to handle stress
BNIP3L	Increases to handle stress
**Assembly factors**	
COX10	Needed for complex IV assembly
COX16	Needed for complex IV assembly
NDUFAF1	Needed for complex I assembly
SDHAF1	Needed for complex II assembly
SDHAF2	Needed for complex II assembly
NDUFAF3	Needed for complex I assembly
NDUFAF4	Needed for complex I assembly
NDUFAF5	Needed for complex I assembly
NDUFAF7	Needed for complex I assembly

Table S3 List of Genes Used to Derive Cellular Quiescence Scores

**Quiescence gene set**
CDKN1A
CDKN1B
BTG1
PTEN
TOB1
TSC1
TSC2
TBC1D7
STK11
FOXO1
FOXO3
FOXO4
KLF2
FOXP1
BACH2
SATB1
PDCD1
CTLA4
TIGIT
VSIR
STAT1

Table S4 List of Antibodies and Markers in the Transcription Factors Panel for PBMC

Marker	Color	Clone	Cat numb	Maker
CD45RA	APC-H7	5H9	561212	BD
CD3	BUV805	SP34-2	568354	BD
CD4	BUV395	L200	564107	BD
CD95	BV605	DX2	305628	Biolegend
CD28	FITC	CD28.2	302906	Biolegend
CD62L	BV711	SK11	565040	BD
TIGIT	eFluo450/BV421	MBSA43	48-9500-41	Invitrogen
BCL6	BUV615	K112-91	568060	BD
RPS6 Ser 244	PCPCyanine5.5	A18024A	935710	Biolegend
TOX	PE	TXRX10	12-6502-82	Invitrogen
EOMES	PE-eFluor610	Dan11mag	61-4875-82	Invitrogen
pMTOR	PE-Cyanine7	MRRBY	25-9718-42	Invitrogen
TCF1	Alexa Fluor647	S33-966	566693	BD
BLIMP1	R718	6D3	567764	BD

Table S5 List of Antibodies and Markers in the Rectal Biopsies High-dimensional Spectral Panel

Marker	Color	Clone	Cat numb	Maker
CD4	BUV395	L200	564107	BD
CD20	BUV496	2H7	569672	BD
CD8	BUV563	RPA-T8	612914	BD
CD64	BUV615	10.1	752365	BD
CCR7	BUV661	2-L1-A	749824	BD
CD16	BUV737	3G8	612786	BD
CD3	BUV805	SP34-2	568354	BD
CD103	Super Bright™ 436	B-Ly7	62-1038-42	Biolegend
CX3CR1	BV510	2A9-1	341622	Biolegend
HLA-DR	BV570	L243	307638	Biolegend
CD69	BV605	FN50	310938	Biolegend
CD25	BV650	BC96	302634	Biolegend
CD45	BV711	D058-1283	740809	BD
CD45RA	BV750	5H9	747465	BD
CD62L	BV786	SK11	565311	BD
CD28	FITC	28.2	302906	Biolegend
CD101	PCPCy5.5	BB27	331016	Biolegend
CD95	PE-CF594	DX2	305634	Biolegend
PD-1 (CD279)	PE-Cy5	eBioJ105	15-2799-42	Thermo Fisher
a4b7	APC	Act-1	NA	NHP
NKG2A	PE-Vio770	REA110	130-113-567	Mylteny
NKp30/CD337	APC/Fire™ 750	P30-15	325226	Biolegend
Ki67	BV480	B56	566109	BD
FoxP3	AF532	PCH101	58-4776-42	Thermo Fisher
GRZB	RB780	GB11	568705	BD
TCF-1	PE	S33-966	564217	BD
Blimp-1	R718	6D3	567764	BD
